# Antiviral and Immunomodulatory Effects of *Pelargonium sidoides DC.* Root Extract EPs® 7630 in SARS-CoV-2-Infected Human Lung Cells

**DOI:** 10.3389/fphar.2021.757666

**Published:** 2021-10-25

**Authors:** Jan Papies, Jackson Emanuel, Nicolas Heinemann, Žarko Kulić, Simon Schroeder, Beate Tenner, Martin D. Lehner, Georg Seifert, Marcel A. Müller

**Affiliations:** ^1^ Institute of Virology, Charité–Universitätsmedizin Berlin, Corporate Member of Freie Universität Berlin, Humboldt-Universität zu Berlin, Berlin, Germany; ^2^ German Center for Infection Research (DZIF), Partner Site Charité, Berlin, Germany; ^3^ Preclinical R & D, Dr. Willmar Schwabe GmbH & Co. KG, Karlsruhe, Germany; ^4^ Department of Paediatric Oncology/Haematology, Otto-Heubner Centre for Paediatric and Adolescent Medicine (OHC), Charité–Universitätsmedizin Berlin, Corporate Member of Freie Universität Berlin, Humboldt-Universität zu Berlin, and Berlin Institute of Health, Berlin, Germany; ^5^ Department of Paediatrics, Faculty of Medicine, University of São Paulo, São Paulo, Brazil; ^6^ Martsinovsky Institute of Medical Parasitology, Tropical and Vector Borne Diseases, Sechenov University, Moscow, Russia

**Keywords:** coronavirus, pelargonium root extract (EPs® 7630), COVID-19, phytomedicine, SARS-CoV-2, antivirals, cytokine storm

## Abstract

Treatment options for COVID-19 are currently limited. Drugs reducing both viral loads and SARS-CoV-2-induced inflammatory responses would be ideal candidates for COVID-19 therapeutics. Previous *in vitro* and clinical studies suggest that the proprietary *Pelargonium sidoides DC.* root extract EPs 7630 has antiviral and immunomodulatory properties, limiting symptom severity and disease duration of infections with several upper respiratory viruses. Here we assessed if EPs 7630 affects SARS-CoV-2 propagation and the innate immune response in the human lung cell line Calu-3. In direct comparison to other highly pathogenic CoV (SARS-CoV, MERS-CoV), SARS-CoV-2 growth was most efficiently inhibited at a non-toxic concentration with an IC50 of 1.61 μg/ml. Particularly, the cellular entry step of SARS-CoV-2 was significantly reduced by EPs 7630 pretreatment (10–100 μg/ml) as shown by spike protein-carrying pseudovirus particles and infectious SARS-CoV-2. Using sequential ultrafiltration, EPs 7630 was separated into fractions containing either prodelphinidins of different oligomerization degrees or small molecule constituents like benzopyranones and purine derivatives. Prodelphinidins with a low oligomerization degree and small molecule constituents were most efficient in inhibiting SARS-CoV-2 entry already at 10 μg/ml and had comparable effects on immune gene regulation as EPs 7630. Downregulation of multiple pro-inflammatory genes (*CCL5*, *IL6*, *IL1B*) was accompanied by upregulation of anti-inflammatory *TNFAIP3* at 48 h post-infection. At high concentrations (100 μg/ml) moderately oligomerized prodelphinidins reduced SARS-CoV-2 propagation most efficiently and exhibited pronounced immune gene modulation. Assessment of cytokine secretion in EPs 7630-treated and SARS-CoV-2-coinfected Calu-3 cells showed that pro-inflammatory cytokines IL-1β and IL-6 were elevated whereas multiple other COVID-19-associated cytokines (IL-8, IL-13, TNF-α), chemokines (CXCL9, CXCL10), and growth factors (PDGF, VEGF-A, CD40L) were significantly reduced by EPs 7630. SARS-CoV-2 entry inhibition and the differential immunomodulatory functions of EPs 7630 against SARS-CoV-2 encourage further *in vivo* studies.

## Introduction

The ongoing SARS-CoV-2 pandemic has presented challenges to health systems worldwide due to the limited availability of vaccines and effective antiviral drugs. The upper respiratory tract is the primary location of SARS-CoV-2 replication, especially during the early phase post-infection (Wolfel et al., 2020). Efficient virus replication in the nose, mouth, throat, and trachea was observed facilitating human-to-human transmission. Common symptoms of infection include fever, cough, and anosmia (Grant et al., 2020). Up to 17% of infected individuals develop more severe symptoms requiring hospitalization and up to 29% of severe cases need intensive care support (Gottlieb et al., 2020; Myers et al., 2020; Wang et al., 2020; Rosas et al., 2021). Rapid disease progression results in severe COVID-19, which is accompanied by overt immunological dysregulations (Ghazavi et al., 2021; Petrey et al., 2021). In particular, the cytokines IL-6, IL-8, CXCL9, and CXCL10 (IP-10) were identified as putative prognostic disease progression markers for COVID-19 (Li et al., 2020; Sugiyama et al., 2021). Moreover, growth factors like PDGF, FGF-2, VEGF-A were strongly associated with hospitalization and the onset of critical disease (Yeung et al., 2016; Pang et al., 2021; Petrey et al., 2021). Consequently, anti-inflammatory, immunosuppressant drugs like dexamethasone are the current state-of-the-art treatment options for COVID-19, generally administered in the second week post-onset of disease (Group et al., 2021). Unfortunately, SARS-CoV-2-specific antiviral drugs have shown limited effects in large clinical trials, which might partially be associated with delayed administration when virus replication has already waned (Consortium et al., 2021; Group et al., 2021; Zhang and Mylonakis, 2021). In addition, translation of *in vitro* findings is challenged by limited tissue accessibility of compounds to the respiratory tract or suboptimal pharmacokinetics (Wienkers and Heath, 2005; Agu and Ugwoke, 2011; Yang et al., 2018; Henise et al., 2019). Accessible and well-tolerated drugs with both antiviral and immunomodulatory effects, which are orally applicable or inhalable, would be ideal candidates for clinical investigations. Early reduction of viral loads might limit immunological dysregulation, severe disease progression, and human-to-human transmission.

Considering clinical safety and availability, herbal preparations with antiviral and/or immunomodulatory activities might qualify for repurposing in the context of COVID-19 (Hensel et al., 2020; Silveira et al., 2020; Brendler et al., 2021). Multiple double-blind, randomized, placebo-controlled clinical trials have reported that the *Pelargonium sidoides DC.* (Geraniaceae) root extract EPs 7630 (EPs^®^ 7630 is a proprietary extract and active ingredient in pharmaceuticals manufactured by Dr. Willmar Schwabe GmbH and Co. KG.) is efficacious as a treatment for acute bronchitis (Matthys et al., 2003; Chuchalin et al., 2005; Matthys and Funk, 2008; Kamin et al., 2010a; Kamin et al., 2010b; Matthys et al., 2010; Kamin et al., 2012), sinusitis (Bachert et al., 2009), common cold (Lizogub et al., 2007; Riley et al., 2018; Riley et al., 2019), and acute non-GABHS tonsillopharyngitis (Bereznoy et al., 2003). Similar to other proanthocyanidin-containing substances such as green tea (Chourasia et al., 2021; Zhang et al., 2021), *in vitro* data suggest that EPs 7630 has broad-spectrum antiviral and antibacterial activity as well as immunomodulatory effects, although the precise mechanism of action has not been identified (Moyo and Van Staden, 2014). Distinct antiviral effects of EPs 7630 have been observed in concentrations up to 100 μg/ml, with activity reported mainly against enveloped viruses such as influenza A virus (IAV; e.g. H1N1, H3N2), respiratory syncytial virus (RSV), parainfluenza virus, and HCoV (HCoV-229E) (Michaelis et al., 2011; Theisen and Muller, 2012). Impaired viral hemagglutination and reduced neuraminidase activity might inhibit IAV entry and release of viral particles (Theisen and Muller, 2012). Although the distinct contributions of the individual constituents of EPs 7630 are still not fully defined, polyphenolic compounds, in particular prodelphinidins, might be responsible for the described antiviral effects (Michaelis et al., 2011; Theisen and Muller, 2012).

In addition to virus inhibition, different immunomodulatory effects of EPs 7630 were reported from non-clinical and clinical studies with evidence for both enhancement of host defense mechanisms and attenuation of inflammatory responses, respectively. This suggests that selective effects depend on the applied preclinical model or the clinical context. EPs 7630 stimulates nitric oxide release (NO) and induces antiviral type I interferon and different cytokines such as IL-22 (Kolodziej and Kiderlen, 2007; Thale et al., 2011; Witte et al., 2015; 2020). In contrast, EPs 7630 pretreatment attenuated LPS-induced sickness behavior in mice, which indicates an anti-inflammatory effect in this setting of excessive sterile inflammation (Noldner and Schotz, 2007). In patients with acute bacterial rhinosinusitis, EPs 7630-induced improvement in symptom scores was associated with a reduction of nasal secretion chemokine levels of IL-8, CCL3, and ENA-78, whereas CXCL10 and CCL2 levels were increased compared to the control group, suggesting selective immunomodulatory effects of EPs 7630 during acute respiratory infections (Peric et al., 2021). Based on previous reports on clinical efficacy and safety of EPs 7630 in treating acute respiratory tract infections from more than 30 clinical trials (Schapowal et al., 2019; Brendler et al., 2021) and the antiviral and immunomodulatory activities described above, we employed a set of different *in vitro* experiments to assess the potential effects of EPs 7630 in the context of experimental SARS-CoV-2 infection in human lung cells.

## Materials and Methods

### *Pelargonium sidoides DC.* Extract EPs 7630 and Extract Fractionation

For all experiments, a sample of a production batch (EXCh. 878) of EPs 7630, an extract of *Pelargonium sidoides DC.* roots (1:8–10), dried, extraction solvent: ethanol 11% (w/w) and fractions thereof were used. Roots of *Pelargonium sidoides DC.* were collected in South Africa (e.g., Eastern Cape). The dried material was tested in an array of DNA-based and phytochemical methods to confirm the quality and identity of the herbal material. Pharmacognosy was done by the quality control department of Dr. Willmar Schwabe GmbH and Co. KG. Voucher specimens of every lot are deposited in the Department of Pharmacognosy to be retained for 10 years.

For fractionation, 10 g of EPs 7630 extract was dissolved in 200 ml of an aqueous 15% ethanol (v/v) solution and fractionated by ultrafiltration, using a 50 ml Merck Millipore Amicon stirred cell with compatible Ultracel filter discs. The fractionation was carried out in a serial fashion, beginning with ultrafiltration through a filter disc with a cut-off of 30 kDa. The 200 ml extract solution was applied to the stirred cell in portions and concentrated to about 10–20 ml. Subsequently, it was washed with 3 × 30 ml aqueous 15% ethanol (v/v) solution. The retentate was collected, ethanol was evaporated on a rotary evaporator and the resulting aqueous solution was freeze-dried for further use. The filtrate of the ultrafiltration was also collected and applied to the next serial fractionation step with a filter disc of 10 kDa cut-off. The aforementioned procedure was repeated with filter discs with cut-offs of 5 kDa, 3 kDa, and 1 kDa, resulting in dry fractions of >30 kDa, 10–30 kDa, 5–10 kDa, 3–5 kDa, 1–3 kDa, and <1 kDa.

### Molecular Characterization of Extract Fractions by HPLC, Gel Permeation Chromatography, and NMR

The HPLC-UV chromatograms were recorded on a Thermo Vanquish UHPLC coupled to a diode array detector (DAD). For separation, a Waters Atlantis T3 (3 μM, 2 × 150 mm) column without pre-column was used. Eluent A consisted of 2.5% (v/v) acetonitrile and 0.5% (v/v) formic acid in water. Eluent B consisted of 5% (v/v) water and 0.5% (v/v) formic acid in acetonitrile. The separation parameters were as follows: 0.2 ml/min flow rate, column temperature of 25°C, UV detection wavelength of 280 nm, and injection volume of 4 µl of a 5 mg/ml *Pelargonium sidoides DC.* extract EPs 7630 and 2 µl of 10 mg/ml ultrafiltration fractions, all dissolved in an aqueous 40% acetonitrile solution. The gradient was as follows: from 0.0–10.0 min linear from 0 to 5% Eluent B, from 10.0–65.0 min linear from 5 to 50% Eluent B, from 65.0 to 66.0 min linear from 50 to 100% Eluent B, from 66.0 to 71.0 min isocratic 100% Eluent B column wash, from 71.0–72.0 min linear from 100 to 0% Eluent B followed by 8 min equilibration period with 0% Eluent B, resulting in a total run time of 80.00 min.

Analysis of the chromatogram was performed using ACDLabs Spectrus Processor Software v2017.2.1. The fractions were complementarily characterized using NMR spectroscopy and gel permeation chromatography. NMR spectra were acquired on a Bruker Avance III HD System equipped with an inverse TCI Prodigy Cryoprobe. The Larmor frequencies for ^1^H and ^13^C were 600 and 150 MHz, respectively. The samples >1 kDa were dissolved at concentrations of 20 mg in 600 µl DMSO-d_6_. The sample <1 kDa was not soluble in DMSO-d_6_ and D_2_O was used for this sample instead with the same concentration of 20 mg in 600 µl. For referencing, tetramethylsilane, or trimethylsilylpropionic acid-d_4_ were used, respectively, and all spectra were acquired with a sample temperature of 25°C. 1D-^1^H spectra were acquired with 32 accumulated scans with a spectral width of 18 ppm, a transmitter frequency offset of 8 ppm, and a digital resolution of 64 k data points. The spectra were processed with an exponential window function with a line broadening of 0.3 Hz. ^1^H-^13^C-HSQC spectra were acquired with 16 accumulated scans, 256 increments for the ^13^C dimension, and 1730 data points for the ^1^H dimension. The spectral widths were 12.0 ppm at 5.5 ppm transmitter offset and 186.0 ppm at 88.0 ppm transmitter offset for ^1^H and ^13^C, respectively. The spectra were processed with 1 k data points and a sine square window function with a sine bell shift of 2 for both dimensions. The spectra were recorded and processed with Bruker Topspin software (v3.6pl7) and analyzed with ACD/Labs Spectrus Processor (v2017.2.1).

Gel permeation chromatography was carried out on a Hitachi LaChrom system, coupled to a DAD. For separation, a Polymer Standards Service MCX analytical 100 Å column (5 μm, 8 × 300 mm) with a total run time of 30 min and an isocratic elution of an aqueous 40% vol. acetonitrile solution with a flow rate of 0.8 ml/min was used. The column temperature was 50°C and signals were detected by UV at 230 nm. The injection volume was 10 μl at a sample concentration of 4.5 mg/ml in elution solvent. For calibration of the retention times, epicatechin 0.8 mg/ml (monomer), procyanidin B2 1.0 mg/ml (dimer), and procyanidin C1 0.8 mg/ml (trimer) were used, respectively. Standards for higher oligomeric proanthocyanidins were not commercially available. The molecular weight of the sample was estimated by extrapolating the elution volumes of the mono, di and trimer.

### Cell Lines

VeroFM (ATCC CCL-81), VeroE6 (ATCC CRL-1586), and Calu-3 (ATCC HTB-55) cells were grown in Dulbecco’s Modified Eagle’s Medium (DMEM) supplemented with 10% fetal bovine serum (FBS), 1% penicillin/streptomycin, 1% non-essential amino acids, and 1% sodium pyruvate at 37°C and 5% CO_2_. All cell lines were cultivated under sterile laboratory conditions and tested for simian virus 5 and *mycoplasma* contamination.

### Virus Strains and Infection

The SARS-CoV-2 strain Munich/2020/984 (BetaCoV/Munich/BavPat1/2020) was isolated from a respiratory swab obtained from the early 2020 Munich patient cohort (GenBank: MT270101; GISAID: EPI_ISL_406,862). For virus infection with SARS-CoV-2, 2x10e5 to 3x10e5 cells/ml were seeded in 6-well plates or 24-well plates. After 24 h, cells were infected with SARS-CoV-2 in a serum-free medium. After 1 h, virus dilutions were removed, and the wells were washed twice with PBS and refilled with DMEM (supplemented as described previously). Samples were taken at the indicated time points. Infection with SARS-CoV (isolate Hong Kong/6109) (Hattermann et al., 2005) and MERS-CoV (Human betacoronavirus 2c EMC/2012; Genbank: JX869059.2), as well as SARS-CoV-2 Alpha (BetaCoV/Baden-Wuerttemberg/ChVir21528/2020; EPI_ISL_754,174) and Beta (Baden-Wuerttemberg/ChVir22131/2021; EPI_ISL_862,149) variants were performed in an analogous procedure using the same multiplicity of infection (MOI). All virus infection experiments were conducted under biosafety level 3 conditions with enhanced respiratory personal protection equipment.

### Synchronized Infection and Virus Entry Evaluation

Calu-3 and VeroE6 cells were seeded in 24-well plates at a seeding density of 3x10e5 and 2x10e5 cells/well, respectively. 2 h before virus infection, cells were washed once with PBS and pretreated with DMEM containing the indicated substances or DMSO as vehicle control for niclosamide. Virus infections were performed on ice and cells were transferred to 4°C for 30 min after the addition of virus inoculum to allow virus attachment without receptor-mediated virus entry. Cells were subsequently washed five times with cold PBS to remove unbound virus particles. For 0 h p. i. samples, the infected cells were immediately lysed in external lysis buffer (Roche). For 4 h p. i. samples, infected cells were further incubated at 37°C in DMEM containing the same substances and concentrations used for pretreatment. RNA isolation and RT-qPCR were performed as described earlier (Dagotto et al., 2021; Schroeder et al., 2021). For viral subgenomic mRNA (sgmRNA) quantification of the N gene, an MOI of 1 was used.

### Plaque Titration Assays

SARS-CoV-2, mumps virus, and Rift Valley fever virus clone 13 (Muller et al., 1995) plaque-forming units (PFU) were quantified by plaque titration on VeroE6 cells as described before (Dulbecco, 1952; Herzog et al., 2008; Forcic et al., 2010). Briefly, monolayers of VeroE6 cells were seeded in 24-well plates with ∼90% confluency and washed with PBS, incubated with serial dilutions of SARS-CoV-2-containing cell culture supernatants, and overlaid with 1.2% Avicel in DMEM 24 h after seeding. 72 h post-infection, cells were fixed with 6% formalin and visualized by crystal violet staining. The assay-specific cut-off is 10 PFU/ml. All titration experiments were performed in duplicate wells.

### Reverse-Transcription Quantitative PCR

Cellular RNA of diverse immune genes was quantified by RT-qPCR using the Superscript III OneStep RT-PCR (Thermo Fisher Scientific) kit and the oligonucleotides listed in Supplementary Table S1. Total RNA was isolated from compound-treated or SARS-CoV-2-infected Calu-3 cells by automated pipetting using the MagNA Pure 96 extraction system (Roche). Gene expression was calculated relative to *TBP* reference gene expression using the ΔΔCT method (Livak and Schmittgen, 2001). SARS-CoV-2 N gene sgmRNA was quantified in RNA extracts from infected VeroE6 and Calu-3 cells using RT-qPCR and *TBP* as reference gene. Gene expression was calculated relative to *TBP* reference gene expression using the ΔΔCT method (Livak and Schmittgen, 2001). To monitor virus growth, SARS-CoV-2 RNA was quantified in cell culture supernatants by RT-qPCR targeting the SARS-CoV-2 E gene, as described before (Corman et al., 2020).

### Vesicular Stomatitis Virus-pseudo-particle Assay

SARS-CoV-2 spike (S) protein-dependent viral entry was assessed using an established vesicular stomatitis virus (VSV) pseudo-particle (VSVpp) assay as described elsewhere (Kleine-Weber et al., 2019; Hoffmann et al., 2020a; Zettl et al., 2020). Briefly, VeroFM and Calu-3 cells were seeded in DMEM in 96-well plates with a density of 50–70% 24 h before infection with VSVpp. For pretreatment, medium was removed 2 h before infection and replaced by fresh DMEM containing EPs 7630, fractions of EPs 7630, camostat mesylate, niclosamide, or DMSO as vehicle control for niclosamide in indicated concentrations. After pre-incubation, medium was removed and fresh DMEM containing VSVpp carrying the SARS-CoV-2 spike (BetaCoV/Munich/BavPat1/2020) or pCG1 vector control was added to the cells. Plates were then centrifuged for 30 min at 4°C and 300 x g to achieve synchronized infection. After additional incubation for 90 min at 37°C, 5% CO_2_, compound-containing medium was added to the cells. To measure luciferase production, which correlates with successful viral entry, cell lysates were prepared after 16 h (VeroFM) or 24 h (Calu-3) using passive lysis buffer (Promega). Lysates were then transferred to opaque 96-well plates and luminescence was measured in a multi-well plate reader (Berthold) using Luciferase Assay Substrate (Promega) according to the manufacturer’s recommendations.

### Cytokine Quantification

To assess cytokine levels in Calu-3 cell supernatant, 25 µl of supernatant were sampled before infection and at 8 and 48 h post-infection with SARS-CoV-2. Cytokines were quantified using a Human Cytokine/Chemokine/Growth Factor Panel A 48-Plex Premixed Magnetic Bead Multiplex Assay (Merck Millipore), using the Luminex MAGPIX System in 96-well plate format, according to the manufacturer’s instructions. Plate washing steps were performed using the HydroFlex Microplate Washer (Tecan). Calibration and verification checks were met for all of the analytes. Analytes for which quality controls were out of the expected range or for which standard curves exhibited an *R* ^2^ value of <0.80 were excluded from the dataset. All remaining analytes had standard curves with *R* ^2^ values > 0.90, except for IL-9 (*R* ^2^ = 0.84).

### Cell Viability Assay

The viability of EPs 7630- or fraction-treated VeroFM and Calu-3 cells was assessed using the CellTiter-Glo 2.0 Cell Viability Assay (Promega) according to the manufacturer’s instructions. Briefly, cells were seeded in 96-well plates and treated with the indicated concentrations of EPs 7630 or ultracentrifugation fractions. After 48 h cells were lysed and the luminescence signal was measured using a multi-well plate reader (Berthold). Viability was calculated in relation to untreated cells and reported as percent of control.

## Results

### EPs 7630 Inhibits Propagation of Highly Pathogenic Coronaviruses in Non-cytotoxic Concentrations

**FIGURE 1:**
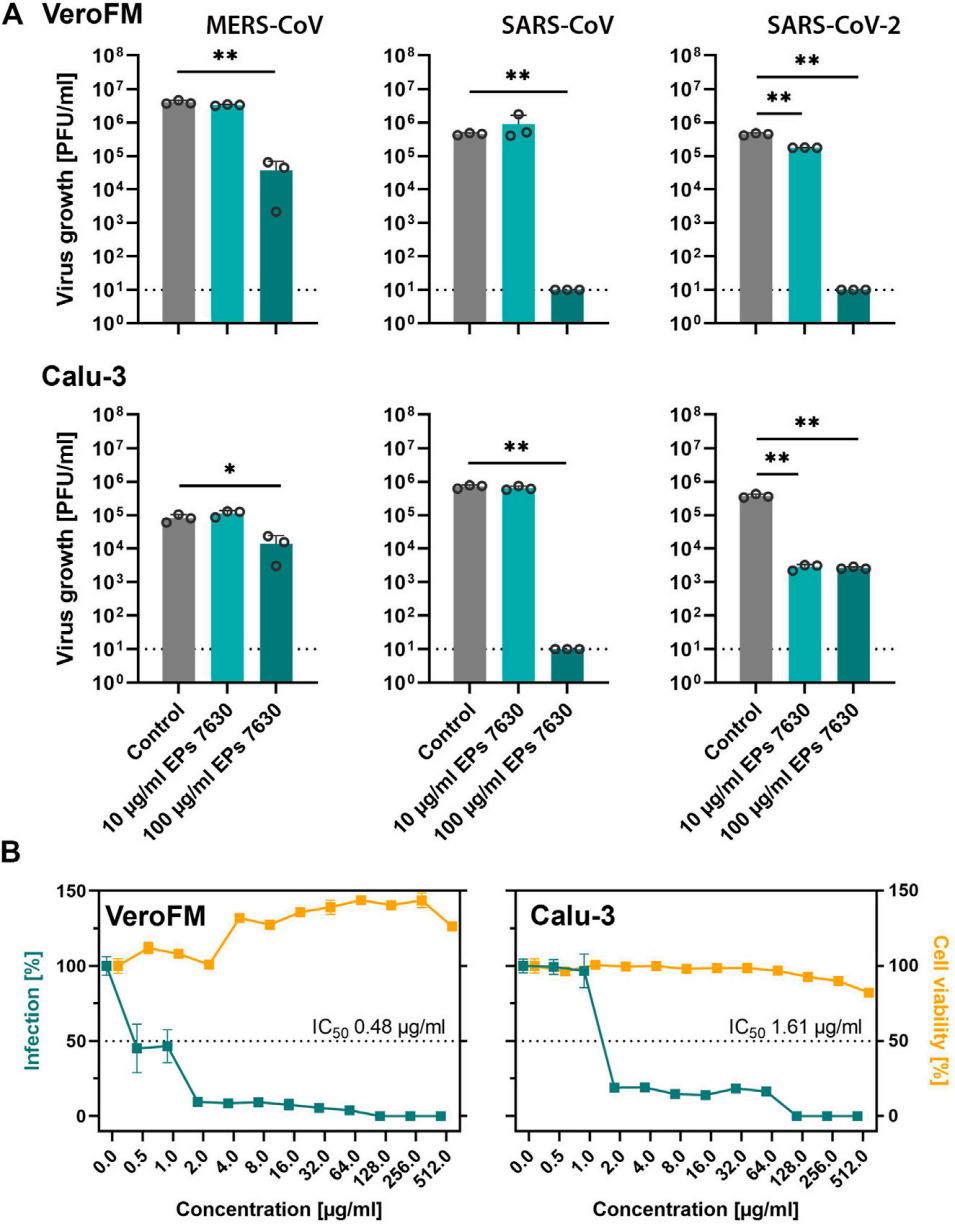
EPs 7630 inhibits the propagation of highly pathogenic coronaviruses. **(A)** Comparison of virus growth of SARS-CoV, MERS-CoV, and SARS-CoV-2 in VeroFM and Calu-3 cells using an MOI of 0.0005 and indicated concentrations of EPs 7630. Virus-containing supernatants were collected 48 h post-infection and viral titers were determined as plaque-forming units (PFU)/ml by plaque titration assay. Bars represent mean values and SD from *n* = 3 biological samples. Statistical significance (treatment vs control) is indicated by (*) as determined by unpaired *t*-test with Welch´s correction. (*) = *p*<0.05; (**) = *p*<0.01. Control = medium only. **(B)** SARS-CoV-2 IC50 determination (plaque titration) and cell viability assay (CellTiter Glo 2.0) in VeroFM and Calu-3 cells at 48 h post-treatment/-infection. Data are shown as percent of untreated cells and represent mean values from at least n = 3 biological samples.

Previous studies showed that respiratory viruses such as common cold CoV and Influenza A virus can be inhibited by EPs 7630 treatment *in vitro* (Michaelis et al., 2011; Theisen and Muller, 2012), while *in vivo* treatment of influenza-infected mice with EPs 7630 led to a reduction of viral burden, attenuation of body weight loss, and improved survival (Theisen and Muller, 2012). In patients with common cold, the EPs 7630 treatment outcomes assessed as reduction of symptoms were as favorable in patients with confirmed HCoV infection as in patients with other viral infections (Keck et al., 2021). To explore putative inhibitory *in vitro* effects of EPs 7630 on highly pathogenic CoV (SARS-CoV, MERS-CoV, and SARS-CoV-2), we infected IFN-deficient (VeroFM) monkey kidney cells and IFN competent human lung cells (Calu-3) with a low MOI (0.0005) in the absence and presence of EPs 7630 (10, 100 μg/ml). Infectious virus particles in cell culture supernatants were determined by virus plaque assay at 48 h post-infection. EPs 7630 significantly inhibited propagation of all highly pathogenic CoV at 100 μg/ml at 48 h post-infection by up to >99% (Figure 1A). In the case of SARS-CoV-2, significant growth inhibition was already observed using 10 μg/ml in both cell lines. Since clinical applications and repurposing approaches rely on low inhibitory concentrations, we determined IC50 values for SARS-CoV-2 in both cell lines. The EPs 7630-specific IC50 for SARS-CoV-2 inhibition was 0.48 μg/ml in VeroFM cells and 1.61 μg/ml in Calu-3 cells (Figure 1B, green) being well within non-cytotoxic ranges (Figure 1B, yellow) and highly comparable to previous studies (Michaelis et al., 2011; Walther et al., 2020). To verify the antiviral activity of EPs 7630 against newly emerging SARS-CoV-2 variants, we analyzed its impact on SARS-CoV-2 Alpha and SARS-CoV-2 Beta propagation, both of which were classified as variants of concern (VOC) by the World Health Organization (Abdool Karim and de Oliveira, 2021; Konings et al., 2021). We found substantial inhibition of SARS-CoV-2 VOC propagation, comparable to the original Munich patient isolate in Calu-3 cells using 100 μg/ml EPs 7630 (Supplementary Figure S1A). At low concentrations of 10 μg/ml EPs 7630, we did not detect a significant reduction of viral RNA levels, possibly as a consequence of increased replicative fitness reported for the analyzed VOCs (Pyke et al., 2021; Rosenke et al., 2021; Touret et al., 2021). To exclude unspecific EPs 7630-induced inhibitory effects in Calu-3 cells, virus growth of other enveloped viruses, specifically mumps virus (MuV) and a Rift Valley fever virus (RVFV) clone 13-based reporter virus (Kuri et al., 2010) was tested in the absence and presence of EPs 7630 (Supplementary Figure S1B). Whereas RVFV growth was significantly reduced at treatment concentration 100 μg/ml, mumps virus, in analogy to measles virus (Michaelis et al., 2011), was not inhibited by EPs 7630.

### EPs 7630 Shows Pronounced SARS-CoV-2 Entry Inhibition in TMPRSS2-Positive Calu-3 Cells

**FIGURE 2:**
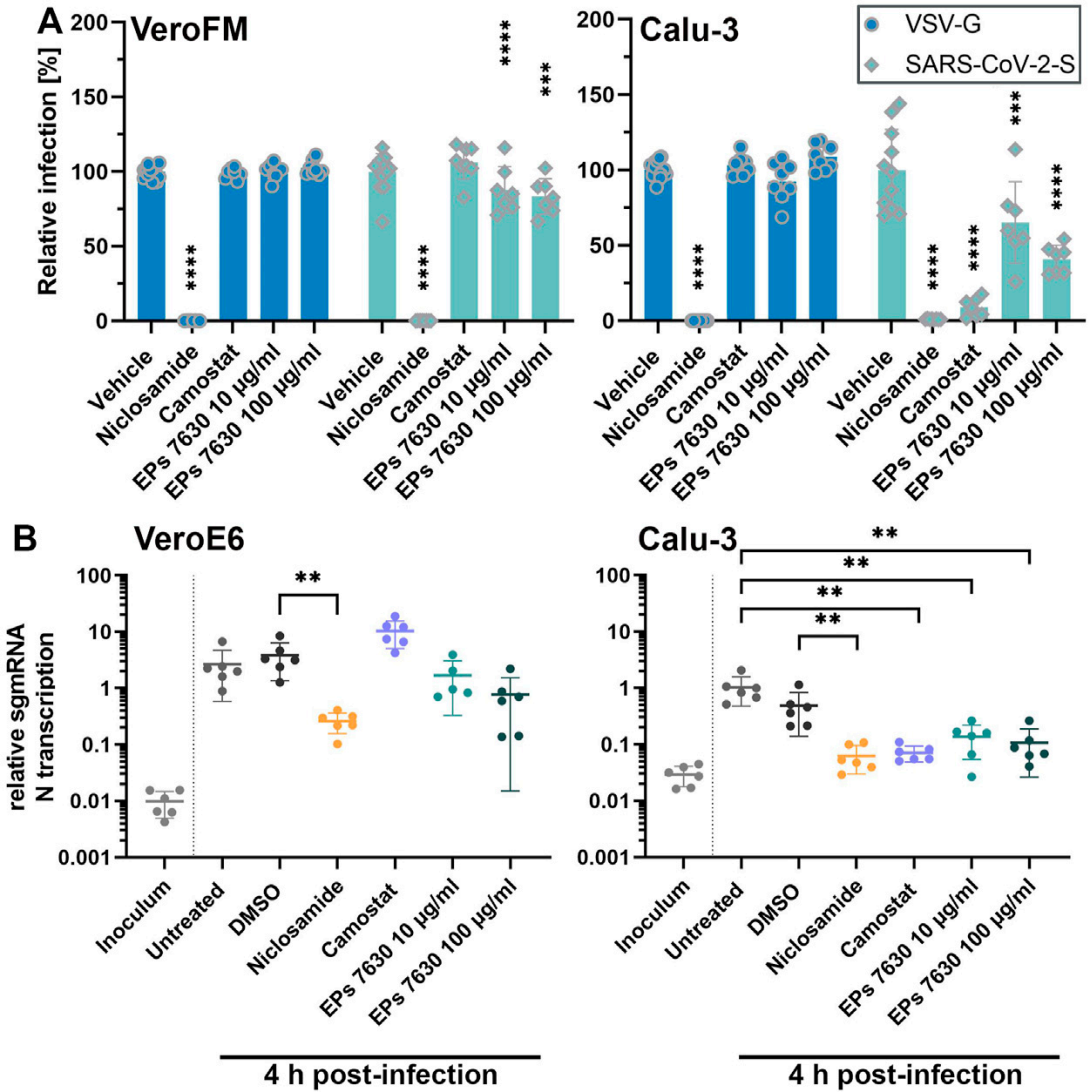
EPs 7630 inhibits entry of VSVpp-SARS-CoV-2-S and SARS-CoV-2 in TMPRSS2-negative VeroFM and TMPRSS2-positive Calu-3 cells. Cells were pre-treated with the indicated compounds for 2 h pre-infection at 37°C. **(A)** Infection with luciferase-producing VSVpp-SARS-CoV-2-Spike (SARS-CoV-2-S), VSV-G control, or pCG1 vector control was done in the presence of compounds for 30 min at 4°C at 300 × g followed by 1.5 h incubation at 37°C to achieve synchronous infection. The medium was then replaced by compound-containing DMEM as indicated. As controls, we applied 10 μM niclosamide (endosomal entry blocker) and 10 μM camostat mesylate (TMPRSS2 inhibitor). Cell lysates were prepared after 16 h (VeroFM) or 24 h (Calu-3) and luminescence was measured using a multi-mode 96-well plate reader. Bars represent mean values and SD from at least *n* = 7 biological samples from two independent experiments. Vehicle = DMSO-(niclosamide) or medium (remaining compounds). Statistical significance (treatment vs. control) is indicated by (*) as determined by two-way ANOVA with Dunnett's multiple comparison testing. (*) = *p* < 0.05; (**) = *p* < 0.01; (***) = *p* < 0.001; (****) = *p* < 0.0001. **(B)** VeroE6 and Calu-3 cells were pretreated with the indicated compound 2 h as described in **(A)**. Infection with SARS-CoV-2 (MOI = 1) was done by incubation for 15 min at 4°C, followed by 30 min at 37°C to achieve synchronized infection. The medium was then replaced by compound-containing DMEM as indicated. As controls, we applied 10 μM niclosamide (endosomal entry blocker) and 10 μM camostat mesylate (TMPRSS2 inhibitor). Cell lysates were prepared 4 h post-infection and subgenomic viral mRNA (sgmRNA) was quantified by RT-qPCR. Data are presented as gene expression relative to the reference gene TBP from n = 6 biological samples from 3 experiments. Statistical significance is indicated by (*) as determined by unpaired t-test. (*) = *p* < 0.05; (**) = *p* < 0.01; (***) = *p* < 0.001; (****) = *p* < 0.0001. DMSO = Dimethyl sulfoxide (vehicle for niclosamide).

As EPs 7630 was shown to inhibit virus entry and release (Theisen and Muller, 2012), we next analyzed the SARS-CoV-2 cellular entry process upon EPs 7630 treatment. SARS-CoV-2 enters cells via ACE2 receptor-mediated endocytosis and direct TMPRSS2-mediated fusion of ACE2-bound SARS-CoV-2 particles with the plasma membrane (Hoffmann et al., 2020a). To model the two entry pathways, we infected TMPRSS2-negative Vero cells (endosomal entry) and TMPRSS2-positive Calu-3 cells (endosomal entry and direct fusion) with SARS-CoV-2 spike (SARS-CoV-2-S) protein-carrying VSV-based pseudo particles (VSVpp). As controls, we applied niclosamide (10 µM), which blocks SARS-CoV-2 replication and possibly restricts endosomal entry (Jurgeit et al., 2012; Prabhakara et al., 2021; Gassen et al., 2021), and camostat mesylate (100 µM), a proven TMPRSS2 inhibitor (Hoffmann et al., 2020a). SARS-CoV-2-S VSVpp entry was efficiently blocked (>98%) by niclosamide in both cell lines (Figure 2A, unprocessed data and controls in Supplementary Figure S2) whereas, expectedly, camostat inhibited VSVpp entry only in TMPRSS2-positive Calu-3 cells. EPs 7630 inhibited SARS-CoV-2-S VSVpp entry more efficiently in Calu-3 cells (59%) as compared to Vero cells (17%) suggesting that the endosomal, as well as the plasma membrane fusion-mediated entry processes, are affected. To further confirm this finding, we analyzed EPs 7630-mediated entry inhibition using infectious SARS-CoV-2 as previously described (Dagotto et al., 2021; Schroeder et al., 2021). Synchronized virus infection of cells with a high MOI (MOI = 1) was followed by PCR-based detection of subgenomic (sg) SARS-CoV-2 nucleocapsid (N) mRNA at 4 h post-infection. Differences in N gene sgmRNA levels at this early time point allowed us to monitor subtle differences in virus entry. In agreement with the VSVpp data, we observed a significant entry inhibition upon niclosamide-treatment in both cell lines, whereas camostat exclusively inhibited SARS-CoV-2 entry in Calu-3 cells (Figure 2B). EPs 7630 clearly showed more efficient entry inhibition in Calu-3 as compared to Vero cells confirming the VSVpp-based data.

### Phytochemical Characterization of *Pelargonium sidoides DC.* Extract EPs 7630 and Its Fractions

EPs 7630 comprises a multitude of molecules including carbohydrates, minerals, peptides, purine derivatives, highly substituted benzopyranones, and oligo- and polymeric prodelphinidins (Schoetz et al., 2008). To identify the chemical substance classes mainly responsible for the observed SARS-CoV-2 entry inhibition, we generated extract fractions by sequential ultrafiltration that contain substances with different molecular weight ranges. The fractions generated by ultrafiltration were analyzed using HPLC-UV, gel permeation chromatography (GPC), and NMR spectroscopy. HPLC analysis was used to monitor the purine derivatives and benzopyranones in the fractions according to the LC-MS assignment reported by Roth et al. (Roth et al., 2021). Polymeric and oligomeric prodelphinidins appeared as broad signals in HPLC analysis, whereas carbohydrates were not detectable due to the absence of chromophores. HPLC analysis revealed that the fractionation by ultrafiltration was successful, yielding major amounts of the small molecule fraction (gallocatechin/epigallocatechin, purine derivatives, benzopyranones) in the <1 kDa fraction (Table 2, Supplementary Figure S3). To estimate the oligomerization degree of the prodelphinidins, a GPC analysis was applied, calibrated by epicatechin (monomer), procyanidin B2 (dimer), and procyanidin C1 (trimer). The prodelphinidins appeared as broad signals in GPC as well, representing the distribution of different oligomerization degrees in the fractions. Comparison and extrapolation of the elution times of the calibration substances indicated the following distribution: fraction 1–3 kDa: di-to tetramers, fraction 3–5 kDa: di-to pentamers, fraction 5–10 kDa: tri-to hexamers, and fraction 10–30 kDa: tetra-to about decamers. The fraction >30 kDa consisted mainly of polymers eluting in the void volume (Supplementary Figure S4). 1D and 2D NMR spectroscopy was used for the detection of carbohydrates and solvent residue. 1D NMR analysis demonstrated a major amount of mono- and dimeric carbohydrates in the <1 kDa fraction (Supplementary Figure S5). According to the intensities of the NMR signal patterns, carbohydrates exceeded the amounts of benzopyranones by approximately five-fold, which is consistent with the constituent ratios described previously (Schoetz et al., 2008). An exact quantitative analysis was not pursued, since this is outside the scope of the present study.

**FIGURE 3:**
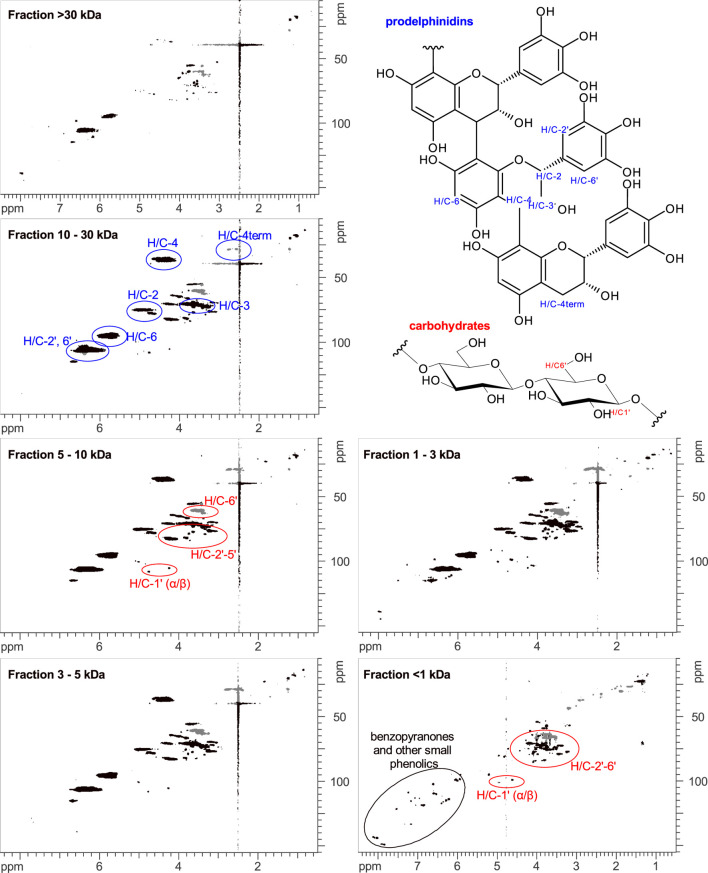
1H-13C-HSQC-NMR spectra of EPs 7630 fractions. Assignments to prodelphinidins (blue), carbohydrates (red), and small phenolics like benzopyranones (black) are color-coded. In the fraction >30 kDa some sharp signals of umckalin (sulfate) and broad signals of polymeric prodelphinidins and carbohydrates are observable. In the fractions 1–30 kDa, oligomeric prodelphinidins and carbohydrates are detectable as broad signals, with only minor amounts of sharp signals of small molecules. In the fraction <1 kDa, only sharp signals of small molecules are detectable, which can be assigned to typical resonances of phenolic and carbohydrate signal patterns. In this <1 kDa fraction, no signals of prodelphinidins are detectable.

The characterization of the oligomerization degree of the prodelphinidin was additionally assessed by 2D NMR spectroscopy. In this regard, the integrals of H/C-4 and H/C-4_term_ in ^1^H-^13^C-HSQC spectra were determined, which represent the number of monomer building blocks that are within the oligomer chain and terminal building blocks, respectively. Since H/C-4_term_ is a methylene group consisting of two protons in contrast to one proton involved in the H/C-4 group, the value of H/C-4_term_ was divided by two and normalized to a value of 1.0. The integrals of H/C-4 were normalized with the same factor as H/C-4_term_. To estimate the mean oligomerization degree, the two normalized integral values were added. In the spectrum of the fraction >30 kDa, the H/C-4 and H/C-4_term_ signals of prodelphinidins had a suboptimal signal-to-noise ratio, and the signals of this fraction were not integrated. The results of the assessment of the oligomerization degree of the fractions 1–30 kDa by NMR analysis are summarized in Table 1. Apart from some minor amounts of small molecules in the 1–3 kDa fraction, the four fractions between 1–30 kDa were largely free of small molecules, consisting mainly of prodelphinidins of different oligomerization degrees and minor amounts of polymeric carbohydrates of undefined oligomerization degree (Figure 3). The >30 kDa fraction contained mainly polymeric prodelphinidins. Interestingly, also umckalin and umckalin sulfate, as well as minor amounts of purine derivatives, were found in this fraction, as shown in the HPLC chromatograms (Supplementary Figure S3). Although not expected by the fractionation strategy, this finding may be due to non-covalent interactions of umckalin (sulfate) and purine derivatives with putative secondary structures of polymeric prodelphinidins or carbohydrates. The results of the phytochemical analyses including a mass balance are summarized in Table 2. All fractions contained small amounts of solvent residue, namely water and ethanol, as detected by NMR analysis, which may contribute to the excess mass (0.2 g) of totaled fractions.

**TABLE 1:** Estimation of prodelphinidin oligomerization degree in EPs 7630 fractions.

Fraction	Relative integral of H/C-4_term_÷2	Relative integral of H/C-4	Resulting mean oligomerization degree
1–3 kDa	1.0	2.55	3.55
3–5 kDa	1.0	3.56	4.56
5–10 kDa	1.0	3.71	4.71
10–30 kDa	1.0	6.14	7.14

**TABLE 2:** Phytochemical characterization of EPs 7630 fractions.

Fraction	Mass balance (g)	Major constituents as determined by HPLC, GPC, and NMR
>30 kDa	4.45	polymeric prodelphinidins, polymeric carbohydrates, umckalin, umckalin sulfate, minor amounts of purine derivatives
10–30 kDa	0.25	higher oligomeric prodelphinidins with a mean oligomerization degree of 7.1, poly-/oligomeric carbohydrates
5–10 kDa	0.23	tri- to hexameric prodelphinidins with a mean oligomerization degree of 4.7, undefined oligomeric carbohydrates
3–5 kDa	0.13	di- to pentameric prodelphinidins with a mean oligomerization degree of 4.6, undefined oligomeric carbohydrates
1–3 kDa	0.16	di- to tetrameric prodelphinidins with a mean oligomerization degree of 3.6, undefined oligomeric carbohydrates, minor amounts of umckalin and umckalin sulfate
<1 kDa	4.98	gallocatechin and epigallocatechin, major amounts of mono/dimeric carbohydrates, purine derivatives, benzopyranones including umckalin and umckalin sulfate

### EPs 7630 Fractions Differentially Inhibit SARS-CoV-2

**FIGURE 4:**
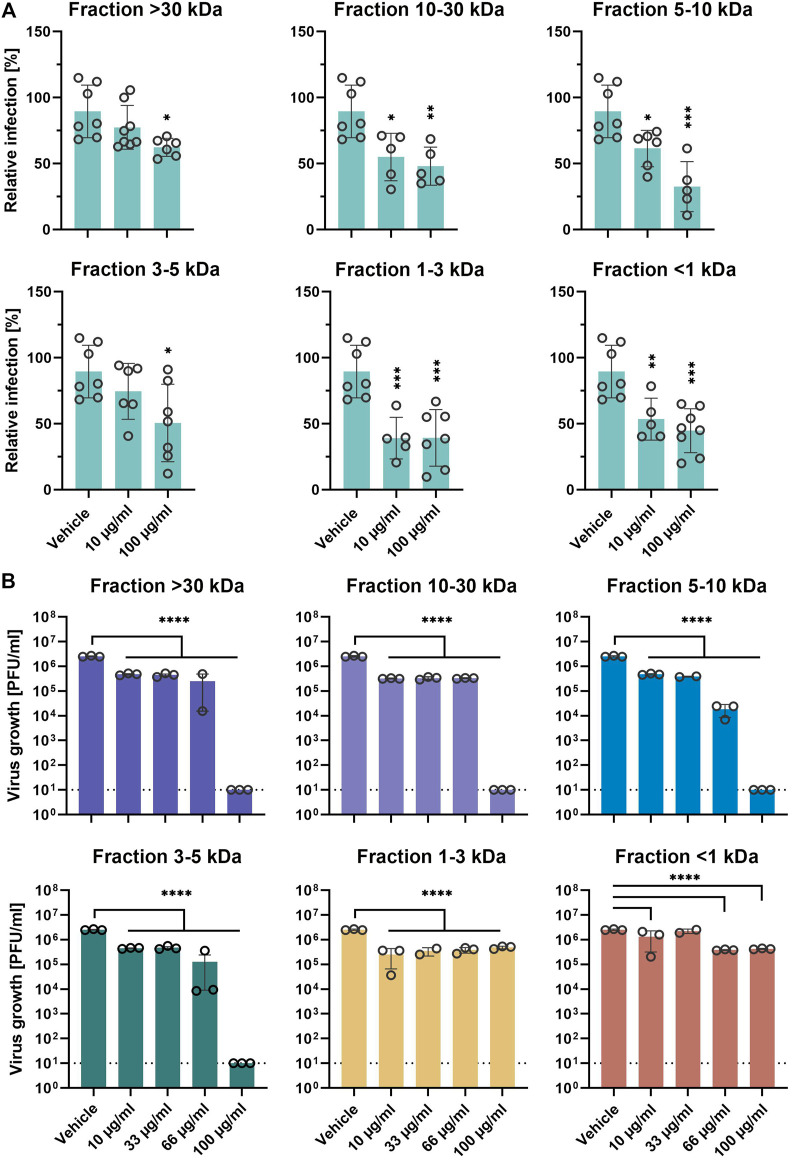
EPs 7630 molecular fractions differentially inhibit SARS-CoV-2 propagation. **(A)** Infection of Calu-3 cells with SARS-CoV-2-Spike VSVpp (SARS-CoV-2-S) or VSV-G as control was done in the presence of compounds for 30 min at 4°C at 500 × g followed by 1-h incubation at 37°C. Cell lysates were prepared after 24 h and the luciferase signal was measured using a multi-mode 96-well plate reader. Bars represent mean values and SD from *n* = 5–8 biological samples from two independent experiments. Technical outliers were removed from the analysis. **(B)** Calu-3 cells were infected with SARS-CoV-2 (MOI = 0.0005) and treated with fractions of ultrafiltrated EPs 7630 simultaneously. Virus-containing supernatants were collected 48 h post-infection and viral titers were determined as plaque-forming units (PFU)/ml by plaque titration assay. Data are derived from *n* = 3 biological samples (for <30 kDa 66 μg/ml, 5–10 kDa 33 μg/ml, 1–3 kDa 33 μg/ml, and <1 kDa 33 μg/ml: *n* = 2). Vehicle medium. Statistical significance (treatment vs. vehicle) is indicated by (*) as determined by two-way **(A)** or one-way **(B)** ANOVA with Dunnett's multiple comparison testing. Asterisks are shown only for significantly different data sets in comparison to vehicle treatment. (*) = *p* < 0.05; (**) = *p* < 0.01; (***) = *p* < 0.001; (****) = *p* < 0.0001.

Next, we aimed to analyze the putative differential inhibitory activity of the 6 different EPs 7630 ultrafiltration fractions on SARS-CoV-2 entry. Pretreated Calu-3 cells were synchronously infected with SARS-CoV-2-S VSVpp in the presence of 10 and 100 μg/ml of the respective fraction during inoculation and post-infection. All fractions showed significant entry inhibition at high concentrations of 100 μg/ml (Figure 4A). Fractions 10–30, 5–10, 1-3, and <1 kDa also showed significant entry inhibition at concentrations of 10 μg/ml, with fraction 1–3 kDa showing the highest efficiency. Confirmatory experiments using infectious SARS-CoV-2 showed significant growth inhibition at 10 μg/ml for all 6 fractions (Figure 4B). Interestingly, medium to high molecular weight fractions (3–5, 5–10, 10–30, and >30 kDa) inhibited virus growth completely at 100 μg/ml whereas low molecular weight fractions 1-3 and <1 kDa reached a maximum inhibition of approximately 90% at 10 and 66 μg/ml, respectively, indicating differential activity of the fractions. Cell viability assays ruled out that cytotoxic effects were responsible for differential inhibition using the indicated concentrations (Supplementary Figure S6).

### EPs 7630 and Distinct Fractions Limit Immune Gene Expression but Enhance Anti-inflammatory TNFAIP3 Induction

**FIGURE 5:**
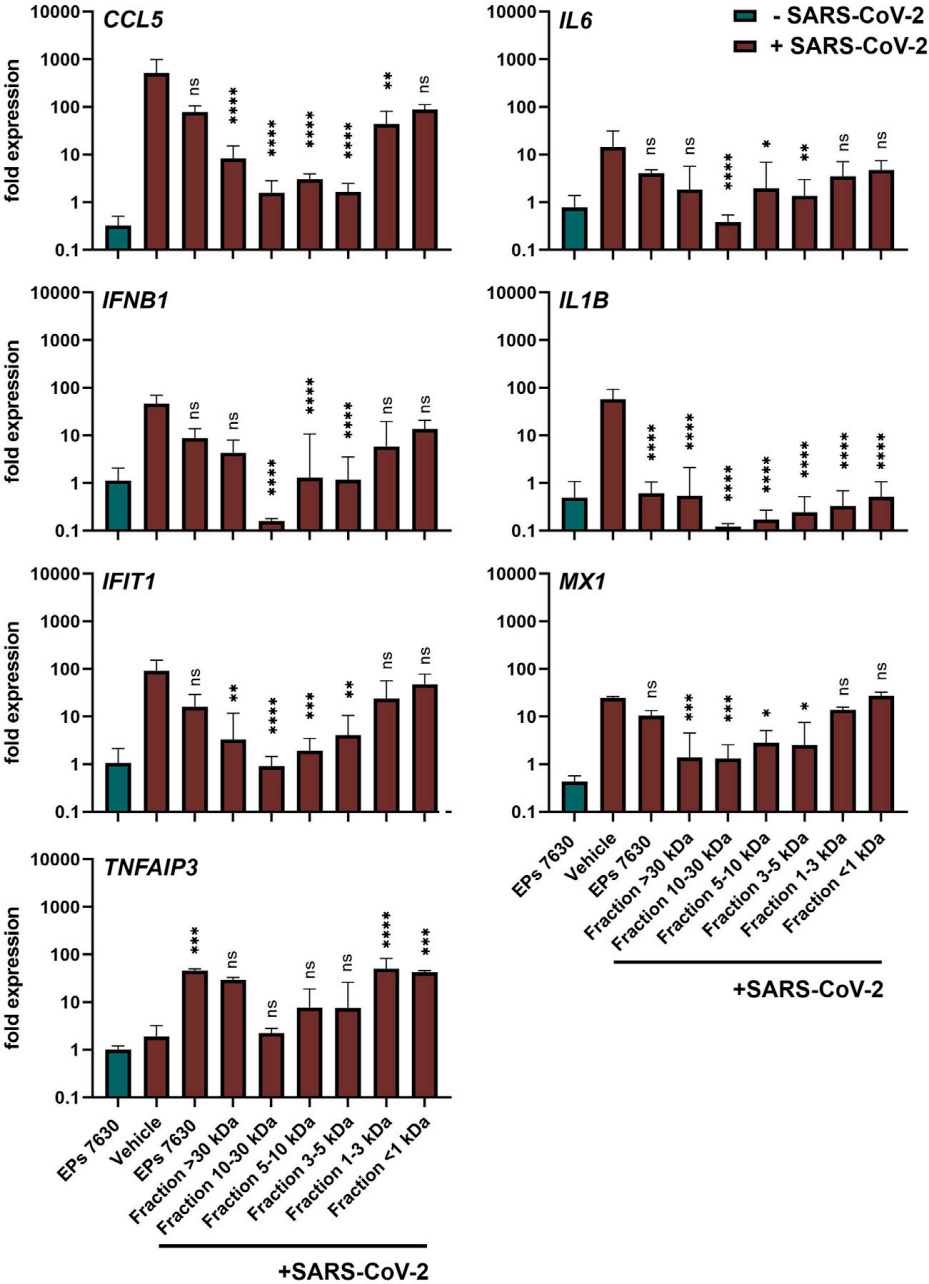
Calu-3 cells treated with EPs 7630 or ultrafiltrated fractions show enhanced anti-inflammatory responses during SARS-CoV-2 infection. Calu-3 cells were treated with EPs 7630 (100 μg/ml), infected with SARS-CoV-2 (MOI = 0.0005; vehicle), or a combination of both. Additionally, cells were treated with ultrafiltrated fractions of EPs 7630 and infected with SARSCoV-2. Cell lysates were prepared 48 h post-infection and cellular RNA of the indicated immune genes was quantified by RT-qPCR. Green bars show EPs 7630-treated, non-infected cells for comparison. Vehicle = medium. Data are derived from *n* = 3 biological samples and are presented as fold gene expression relative to untreated cells and normalized to reference gene expression (*TBP*). Statistical significance in samples from SARS-CoV-2 infected cells (treatment vs. vehicle) is indicated by (*) as determined by two-way ANOVA with Dunnett's multiple comparison testing. (*) = *p* < 0.05; (**) = *p* < 0.01; (***) = *p* < 0.001; (****) = *p* < 0.0001.

Apart from inhibiting virus entry, EPs 7630 has immunomodulatory effects (Noldner and Schotz, 2007; Peric et al., 2021) that might be beneficial for counteracting virus infections and preventing inflammation and immune dysregulation (cytokine storm), which is associated with high COVID-19 morbidity and mortality. The variable SARS-CoV-2 inhibition of the different EPs 7630 fractions (Figure 4B) encouraged us to compare immunomodulatory effects of EPs 7630 and its fractions in SARS-CoV-2-infected Calu-3 cells. Whereas EPs 7630 or its fractions (each 100 μg/ml) alone (Figure 5, green bar; Supplementary Figure S7) had no major effects on pro-inflammatory (*CCL5, IL6, IL1B*), IFN-dependent (*IFNB1, IFIT1, MX1*), or anti-inflammatory (*TNFAIP3*) gene expression, SARS-CoV-2 infection resulted in 10 to 100-fold increased expression of all genes except *TNFAIP3* at 48 h post-infection (Figure 5, red bars). EPs 7630 treatment post-SARS-CoV-2 infection resulted in a significant reduction of *IL1B* gene expression and strong upregulation of anti-inflammatory *TNFAIP3*. All other genes showed a limited but non-significant decrease in gene activation suggesting either limited transcriptional regulation or restoration of mRNA levels late in infection. Notably, reduced virus growth as a consequence of entry inhibition (see Figures 2,4) might also generally limit the SARS-CoV-2-induced upregulation of immune genes. Still, the low molecular weight fractions <1 and 1–3 kDa, which had limited effects on virus propagation using 100 μg/ml (see Figure 4B), resembled EPs 7630-dependent gene regulation patterns with the strongest effects on *IL1B* and *TNFAIP3* during SARS-CoV-2 infection. The 4 fractions between 3 and >30 kDa had minor effects on anti-inflammatory *TNFAIP3*, but strong inhibitory effects on most of the pro-inflammatory and IFN-dependent genes. The differential gene activation patterns might be explained by the composition of the fractions containing gallocatechins, benzopyranones such as umckalin and umckalin sulfate, and purine derivatives in the two low molecular weight fractions, and increasing amounts of di-, tri-, hexa-, oligo-, and polymeric prodelphinidins in the high molecular weight fractions (Table 1).

### EPs 7630 Limits SARS-CoV-2-Induced Inflammatory Response in Calu-3 Cells

The strong EPs 7630-induced inhibition of pro-inflammatory *IL1B* induction and upregulation of anti-inflammatory *TNFAIP3* late post-infection encouraged us to have a closer look at the cytokine secretion profile of epithelial Calu-3 cells. Cytokine production at the primary site of SARS-CoV-2 infection, i.e. the lung epithelium, is crucial for activation of immune cells and priming of inflammatory responses, thereby affecting the outcome of disease (Chua et al., 2020; Mathew et al., 2020; Islam et al., 2021).

**FIGURE 6:**
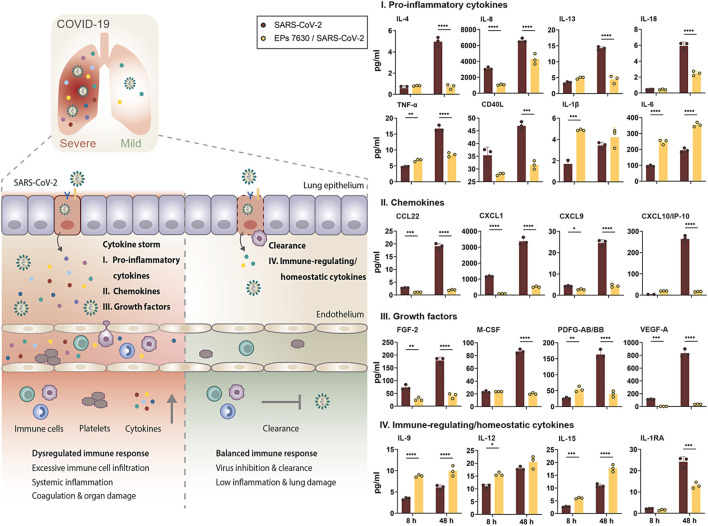
EPs 7630 treatment limits SARS-CoV-2-induced inflammation in Calu-3 lung cells. Supernatants from EPs 7630-treated (100 μg/ml) and EPs 7630-treated and SARS-CoV-2 infected (MOI = 0.0005) Calu-3 cells were analyzed at 8 and 48 h post-infection using the Human Cytokine/Chemokine/Growth Factor Multiplex Assay (Merck Millipore) with the Luminex MAGPIX System according to the manufacturer’s instructions. Data are derived from *n* = 3 biological samples (for IL-1β, CXCL10/IP10: *n* = 2–3). Vehicle = medium only. Statistical significance is indicated by (*) as determined by two-way ANOVA with Tukey’s multiple comparison test. Asterisks are shown only for significantly different data sets. (*) = *p* < 0.05; (**) = *p* < 0.01; (***) = *p* < 0.001; (****) = *p* < 0.0001.

Luminex-based cytokine detection was done early (8 h) and late (48 h) post-SARS-CoV-2 infection with and without EPs 7630 treatment (100 μg/ml). We detected pronounced production of pro-inflammatory cytokines, chemokines, and immunomodulating growth factors in SARS-CoV-2 infected Calu-3 cells, most of which are associated with COVID-19-induced cytokine storm (Figure 6, right panel: I, II, III, red bars) (Cauchois et al., 2020; Donlan et al., 2021; Pang et al., 2021; Petrey et al., 2021; Sugiyama et al., 2021). During the course of infection, the concentration of all analyzed proteins increased at 48 h p. i., analogously to SARS-CoV-2 viral loads in Calu-3 cells (Figure 1). Interestingly, concomitant treatment of infected cells with EPs 7630 strongly reduced the production of distinct cytokine subsets. Protein levels of key inflammatory cytokines like IL-8, IL-13, IL-18, or TNF-α were greatly reduced in supernatants of SARS-CoV-2 infected Calu-3 cells that were simultaneously treated with EPs 7630. In contrast, we noticed an EPs 7630-mediated increase in IL-1β and IL-6 levels early post-infection (8 h), with enhanced IL-6 levels also detectable late after infection (48 h). These effects were also found in EPs 7630-treated Calu-3 cells in the absence of virus infection, highlighting the versatile immunomodulating activities of EPs 7630 beyond cytokine suppression (Supplementary Figure S8).

In the case of chemokines (Figure 6, right panel: II) and growth factors (Figure 6, right panel: III), which coordinate immune cell attraction and infiltration to the site of infection, we consistently found reduced levels in cell supernatants from SARS-CoV-2-infected and EPs 7630-treated cells. Particularly late upon infection (48 h), reduction of secreted proteins ranged from 75.6% reduction for PDGF-AB/BB to 95.8% reduction for VEGF-A. Importantly, chemokines and growth factors that are associated with COVID-19-induced cytokine storm and severe disease progression like CXCL9, CXCL10 (IP10), or VEGF-A exhibited particularly effective EPs 7630-mediated inhibition (CXCL9: 82% reduction; CXCL10: 80.9% reduction) (Costela-Ruiz et al., 2020; Petrey et al., 2021; Sugiyama et al., 2021). Whereas the strongest EPs 7630-mediated cytokine reduction was detected at 48 h p. i., multiple proteins showed significant inhibition in the early phase of SARS-CoV-2 replication at 8 h p. i. (IL-8, TNF-a, CCL22, CXCL1, CXCL9, FGF-2, PDFG-AA/BB, VEGF-A), hinting towards an intrinsic capacity of EPs 7630 to dampen virus-induced inflammation. Indeed, with few exceptions, EPs 7630 was capable of significantly reducing baseline cytokine production in the absence of virus (Supplementary Figure S8, green bars). In contrast, EPs 7630 induced the production of immune-regulating cytokines with a role in inflammation resolution and immune homeostasis in SARS-CoV-2 infected cells (Figure 6, right panel: IV), except for IL-1RA, which correlates with COVID-19 severity and was reduced in our analysis (Zhao et al., 2020). Interestingly, this effect of EPs 7630 treatment was also detected in non-infected cells, boosting the production of immune-regulating cytokines IL-9, IL-12, and IL-15, and potentially setting the course for a more balanced immune response towards virus infection in epithelial cells (Supplementary Figure S8, IV., bottom panel).

## Discussion

Our *in vitro* data show that EPs 7630 blocks SARS-CoV-2 entry, limits SARS-CoV-2 propagation, and differentially regulates immunomodulatory cytokine release. Phytochemical characterization of EPs 7630 fractions identified oligo- and polymeric prodelphinidins as major active antiviral components. Fractions containing small molecular weight constituents such as prodelphinidins of low polymerization degree, benzopyranones, and purine derivatives showed more pronounced immunomodulatory activity on pro-inflammatory *IL1B* and anti-inflammatory *TNFAIP3*. Importantly, the secretion of multiple cytokines and growth factors associated with critical COVID-19 progression was dampened by EPs 7630 in SARS-CoV-2-infected human lung cells.

The broad spectrum of polyphenols such as tannins (Mehany et al., 2021; Umeoguaju et al., 2021) and catechins e.g., from green tea (Chourasia et al., 2021; Zhang et al., 2021) strongly interact with viral envelope proteins and were found to exhibit antiviral activity. To assess the antiviral potential of polyphenol-containing EPs 7630, we infected human lung cells with highly pathogenic human coronaviruses in the presence of EPs 7630. In line with previous studies on HCoV-229E (Michaelis et al., 2011), we detected a strong inhibition of MERS-CoV, SARS-CoV, and SARS-CoV-2 propagation at non-toxic concentrations and low IC50 values for SARS-CoV-2 infection. This observation supports previous findings on cytoprotective and antiviral effects of EPs 7630 and its active ingredients with low cytotoxicity (Conrad et al., 2007; Noldner and Schotz, 2007). In addition, EPs 7630 treatment efficiently reduced SARS-CoV-2 RNA levels in human lung cells infected with VOCs Alpha and Beta, highlighting its broadly-acting antiviral activity and the potential to inhibit newly emerging SARS-CoV-2 variants in the future. Although we detected reduced inhibition of VOC propagation at low concentrations, possibly due to the reported increase of replicative fitness for SARS-CoV-2 Alpha and Beta (Pyke et al., 2021; Rosenke et al., 2021; Touret et al., 2021), the broadly acting antiviral effects of EPs 7630 were clearly retained.

The root extract of *Pelargonium sidoides DC.* is comprised of a multitude of molecules including carbohydrates, minerals, peptides, purine derivatives, highly substituted benzopyranones, and oligo- and polymeric prodelphinidins (Schoetz et al., 2008). The latter contribute to about 40% of the dry extract and comprise manifold structural variety, ranging from monomers to at least 16-mers. Previous studies on the anti-influenza effects of EPs 7630 already suggested that the main activity of the extract depended on the presence of prodelphinidins with oligo- and polymeric structure (Theisen and Muller, 2012). The identification of oligomeric prodelphinidins (i.e., proanthocyanidins) as a major active principle in EPs 7630 aligns with other reports on the inhibition of processes relevant for viral infections and virus-induced host responses such as intracellular signaling (Derksen et al., 2014; Lee et al., 2017; Quosdorf et al., 2017; Tsukuda et al., 2017).

Pronounced antiviral effects were reported against multiple respiratory viruses, including IAV and RSV, while measles virus and several non-enveloped viruses were not affected by EPs 7630 treatment (Michaelis et al., 2011). In the case of *in vitro* IAV infection, mainly prodelphinidins of low to intermediate oligomerization degree contributed to the observed antiviral effect (Theisen and Muller, 2012). The reduced IAV replication was attributed to an EPs 7630-mediated inhibition of hemagglutinin and neuraminidase, which both engage in virus attachment and entry, representing a block of early virus infection. Similarly, other oligomeric proanthocyanidins were shown to block the attachment of herpes simplex virus type-1 or hepatitis B and D virus entry (Gescher et al., 2011; Tsukuda et al., 2017). Here, we found that a part of the antiviral activity of EPs 7630 against SARS-CoV-2 was based on virus entry inhibition. The block of SARS-CoV-2 entry was more pronounced in TMPRSS2-expressing Calu-3 cells than in Vero cells. Although both EPs 7630 and the approved TMPRSS2 inhibitor camostat showed limited efficacy in Vero cells that lack TMPRSS2, residual entry inhibition was detectable for EPs 7630 but not for camostat. Camostat is a known serine protease inhibitor that efficiently blocks SARS-CoV-2 entry by preventing TMPRSS2-mediated SARS-CoV-2 S activation (Hoffmann et al., 2020a). While it was suggested that SARS-CoV-2 entry predominantly relies on TMPRSS2 proteolytic processing in human airway cells, in other cell lines the cysteine protease cathepsin L was reported to participate in TMPRSS2-independent lysosomal SARS-CoV-2 entry (Hoffmann et al., 2020a; Hempel et al., 2021). Flavonoids have been shown to inhibit multiple human serine proteases, but also the cysteine protease cathepsin B, emphasizing their potential as effective protease inhibitors (Ramalho et al., 2015; Xue et al., 2017; Jakimiuk et al., 2021). Although the effects of EPs 7630 on protease activity have not been addressed specifically, flavonoid-based TMPRSS2 inhibition presents a conceivable mechanism for the pronounced SARS-CoV-2 entry inhibition in Calu-3 lung cells. Likewise, in Vero kidney cells that lack TMPRSS2 expression, cathepsin L inhibition by EPs 7630 might explain the TMPRSS2-independent block of SARS-CoV-2 entry that we observed here (Hoffmann et al., 2020b; Matsuyama et al., 2020). Interestingly, the absence of EPs 7630-induced antiviral activity against mumps virus supports the previous finding on measles virus (Michaelis et al., 2011) but contrasts inhibition of respiratory syncytial virus (RSV). Whereas measles and mumps virus mainly use CD150 and CD46 (measles virus) (Hashimoto et al., 2002), or sialic acids and glycan motifs (mumps virus) (Kubota et al., 2016; Kruger et al., 2018; Kubota et al., 2019) for entry, RSV was shown to use distinct cell surface molecules, including glycosaminoglycans (GAG), intercellular adhesion molecule 1 (ICAM-1), and epidermal growth factor receptor (EGFR) (Battles and McLellan, 2019). Previous studies suggested that flavonoids inhibit GAG synthesis (Moskot et al., 2015; Qiu et al., 2017), block ICAM-1 induction (Chen et al., 2004; Owens et al., 2009), and inhibit EGFR (Firdous et al., 2014; Shah and Seth, 2021), potentially explaining the differential antiviral activity of EPs 7630 against RSV and the two paramyxoviruses measles and mumps virus. In line with these reports, it appears conceivable that EPs 7630 targets additional, yet unidentified host cell surface molecules involved in virus attachment, thereby also potentially limiting TMPRSS2-independent entry.

To characterize the components in EPs 7630 that are responsible for SARS-CoV-2 inhibition, we analyzed the effects of EPs 7630 ultrafiltration fractions on virus entry and propagation. Entry inhibition was more pronounced in fractions containing predominantly oligomeric prodelphinidins (fractions 10–30 kDa, 5–10 kDa), but also in low molecular weight fractions (1–3 kDa and <1 kDa). Interestingly, whereas the 1–3 kDa fraction comprises mainly monomeric gallocatechin and prodelphinidin dimers to tetramers with proven effects in virus entry, the <1 kDa fraction contains highly substituted benzopyranones like umckalin and other small molecules. Although little is known about the antiviral effect of these compounds, umckalin and other benzopyranones were shown to modulate immune responses and affect antiviral defense mechanisms (Robertson et al., 2016; Roth et al., 2021). Considering the TMPRSS2-independent entry inhibition that we observed in Vero cells, it appears likely that umckalin and other small molecules contributed to the inhibition of SARS-CoV-2 entry in our study.

To complement our findings on SARS-CoV-2 entry inhibition, we analyzed the antiviral effects of the different fractions on SARS-CoV-2 propagation. Viral inhibition was most pronounced using high to intermediate molecular weight fractions (3–5 kDa - >30 kDa), in contrast to the low molecular weight fractions (1–3 kDa and <1 kDa). These findings corroborate previous reports that identified oligomeric prodelphinidins, which constitute the predominant molecules in these fractions, as the main antiviral ingredient in EPs 7630 (Theisen and Muller, 2012). Previous studies demonstrated that antiviral activity was influenced by prodelphinidin polymer chain length, indicating limited antiviral activity of prodelphinidin mono- and dimers (Conrad et al., 2007; Kolodziej and Kiderlen, 2007). Since these molecules comprise only 3.6% of EPs 7630 total mass, the presented findings hint towards synergistic antiviral effects of different substance classes that take effect in the unfractionated extract. Supporting this hypothesis, we identified limited antiviral efficiency also in high and low molecular weight fractions. Conclusively, the overall antiviral effect that we detected was likely orchestrated by a combination of EPs 7630 active ingredients, which differentially affect virus entry and replication.

In contrast to the antiviral effect mediated by oligomeric EPs 7630 constituents, the fractions <1 kDa and 1–3 kDa displayed a remarkable activation of the major anti-inflammatory factor *TNFAIP3*. Interestingly, both fractions contain the two benzopyranones umckalin and umckalin sulfate. These small molecules may contribute to the anti-inflammatory/immunomodulatory effects that we and others detected for EPs 7630. In addition, we found robust inhibition of pro-inflammatory *IL1B* induction by EPs 7630 and all analyzed fractions. Supporting previous studies on the cytoprotective function of EPs 7630, *IL1B* inhibition might explain the described cytoprotective effects. Considering the cytokine downregulation by EPs 7630, it has to be noted that virus inhibition on its own likely reduces cytokine induction through reduced virus propagation and immune detection. Strikingly, the described effects on *IL1B* and *TNFAIP3* were identified in fractions that showed only limited virus inhibition. These findings suggest immunomodulatory effects independent from virus inhibition and further support the hypothesis of synergistic effects of different molecule classes in EPs 7630.

We broadened our analysis of EPs 7630-based immune modulation by quantifying multiple cytokines and growth factors at the same time in culture supernatants of SARS-CoV-2 infected human lung cells. Epithelial cells of the respiratory tract represent the primary site of SARS-CoV-2 infection, where the virus induces excessive cell death, the production of inflammatory cytokines, and the recruitment of immune cells (Jeyanathan et al., 2020; Islam et al., 2021). Multiple studies reported the massive and dysregulated production of inflammatory cytokines and growth factors in patients with severe COVID-19. In line with previous reports, we detected the production of inflammatory cytokines and growth factors in SARS-CoV-2-infected Calu-3 epithelial lung cells (Callahan et al., 2021; Islam et al., 2021). Of note, key inflammatory factors and drivers of COVID-19-induced cytokine storm were included in our analysis (IL-6, IL-8, IL-13, TNF-α, CXCL9, CXCL10, PDGF, VEGF-A, CD40L, IL1RA) and showed robust upregulation in infected Calu-3 cells (see Figure 6 for graphical overview and details) (Cauchois et al., 2020; Donlan et al., 2021; Pang et al., 2021; Petrey et al., 2021; Sugiyama et al., 2021). Strikingly, EPs 7630 treatment significantly reduced the production of major pro-inflammatory factors in SARS-CoV-2 infected Calu-3 cells. Except for IL-6, all of the aforementioned cytokines showed reduced levels in culture supernatants from infected cells, suggesting an overall reduced inflammatory signature in infected lung epithelial cells. Additionally, we quantified EPs 7630-based cytokine induction in the absence of SARS-CoV-2, corroborating that the observed reduction of inflammatory cytokines was not solely caused by reduced virus growth (for details see Supplementary Figure S8). While the majority of analyzed cytokines were already reduced or unaffected 48 h after EPs 7630 treatment, only IL-6 and the immune-regulating cytokines IL-9, 12, 15 were significantly upregulated. The observed IL-6 stimulation is in line with previous studies reporting EPs 7630-based IL-6 induction (Witte et al., 2015). The impact of IL-6 induction in the context of COVID-19 cytokine storm remains to be addressed in subsequent studies and *in vivo* experiments, but the clear net anti-inflammatory profile of EPs 7630 found in our experiments indicates that strong inflammatory signaling through IL-6 appears unlikely.

Complementing these findings, immune-regulating cytokines with described functionality in maintaining immune homeostasis IL-9, IL-12, IL-15 were upregulated by EPs 7630 treatment alone, but also during SARS-CoV-2 infection. Whereas IL-9 participates in the resolution of inflammation through type 2 innate lymphoid cells, IL-12 was detected in asymptomatic or mild COVID-19 and is suggested to play a key role in protection from cytokine storm and severe disease (Tjan et al., 2021). In addition, IL-15 was reported as a critical immunoregulatory cytokine that maintains immune homeostasis and supports viral clearance (Kandikattu et al., 2020). The finding that EPs 7630 not only limits inflammatory cytokines associated with COVID-19 cytokine storm but also upregulates homeostatic cytokines, underscores the immunomodulating potential of EPs 7630 and its diverse effects on both virus propagation and inflammation.

Previous clinical studies already demonstrated that EPs 7630 has beneficial effects on clinical outcomes of respiratory diseases including common cold CoV. Our *in vitro* study showed an IC50 of 1.61 μg/ml in Calu-3 cells and confirmed that 10–100 μg/ml EPs 7630 have pronounced antiviral and immunomodulatory functions in human epithelial cells encouraging further analysis. *In vivo* studies should clarify if currently used clinical doses of EPs 7630 (3 times per day 20 mg EP 7630 extract) have direct antiviral properties or exert beneficial effects on SARS-CoV-2 infection indirectly through immunomodulation. Since EPs 7630 is well-tolerated and could be applied immediately after a positive test result, its oral application might be beneficial to reduce SARS-CoV-2 replication in the mouth and throat early post-infection (Wolfel et al., 2020; Huang et al., 2021). However, the oral application might limit its potential efficacy for late-stage infections and severe COVID-19 cases where replication mainly takes place in the lower respiratory tract (Rendeiro et al., 2021). In addition, many patients experience loss of smell and pathology-based studies indicated that SARS-CoV-2 replicates in epithelial cells of the olfactory bulb (Meinhardt et al., 2021). The development of a nasal spray or an inhalable formula might enhance its putative antiviral effects. The general feasibility of a topical delivery approach has already been demonstrated in IAV-infected mice, where aerosol delivery of EPs 7630 was shown to improve survival (Theisen and Muller, 2012). A possible limitation of EPs 7630 in the context of SARS-CoV-2 infections might be the observed immunomodulatory effects in Calu-3 cells, especially the increased levels of pro-inflammatory cytokines like IL-6, which is a prognostic marker for severe outcome of disease. However, it should be acknowledged that the location and timing of cytokine production during infection is crucial and we cannot exclude that an early EPs 7630-induced upregulation of such cytokines in epithelial cells of the upper respiratory tract might be beneficial for the disease outcome. The immunological interplay between epithelial and immune cells during virus infections is still quite obscure. Only *in vivo* models might be able to reflect the complexity of virus replication dynamics and immune modulation over time in different cell types and tissues. As EPs 7630 is an approved drug, well-designed clinical studies might resolve the ambiguity of the differential regulation of cytokines on disease outcome.

## Data Availability

The original contributions presented in the study are included in the article/Supplementary files, further inquiries can be directed to the corresponding author.
