# Corrigendum: Antiviral and Immunomodulatory Effects of *Pelargonium sidoides DC.* Root Extract EPs^®^ 7630 in SARS-CoV-2-Infected Human Lung Cells

**DOI:** 10.3389/fphar.2021.814452

**Published:** 2021-12-17

**Authors:** Jan Papies, Jackson Emanuel, Nicolas Heinemann, Žarko Kulić, Simon Schroeder, Beate Tenner, Martin D. Lehner, Georg Seifert, Marcel A. Müller

**Affiliations:** ^1^ Institute of Virology, Charité-Universitätsmedizin Berlin, Corporate Member of Freie Universität Berlin, Humboldt-Universität zu Berlin, Berlin, Germany; ^2^ German Center for Infection Research (DZIF), Partner Site Charité, Berlin, Germany; ^3^ Preclinical R&D, Dr. Willmar Schwabe GmbH & Co. KG, Karlsruhe, Germany; ^4^ Department of Paediatric Oncology/Haematology, Otto-Heubner Centre for Paediatric and Adolescent Medicine (OHC), Charité – Universitätsmedizin Berlin, Corporate Member of Freie Universität Berlin, Humboldt-Universität zu Berlin, and Berlin Institute of Health, Berlin, Germany; ^5^ Department of Paediatrics, Faculty of Medicine, University of São Paulo, São Paulo, Brazil; ^6^ Martsinovsky Institute of Medical Parasitology, Tropical and Vector Borne Diseases, Sechenov University, Moscow, Russia

**Keywords:** SARS-CoV-2, Coronavirus, *Pelargonium sidoides* DC. root extract (EPs^®^ 7630), phytochemical antivirals, drug repurposing, immune modulation, COVID-19, cytokine storm

In the original article, there was a mistake in Figure 2 as published. The legend for Figure 2A was missing in an updated version of the figure after proofs. In addition, a minor mistake was corrected in Figure 6 (CXCL1 y-axis tick numbers partially covered by neighboring graph). The corrected Figure 2 and Figure 6 appear below.

**FIGURE 2:**
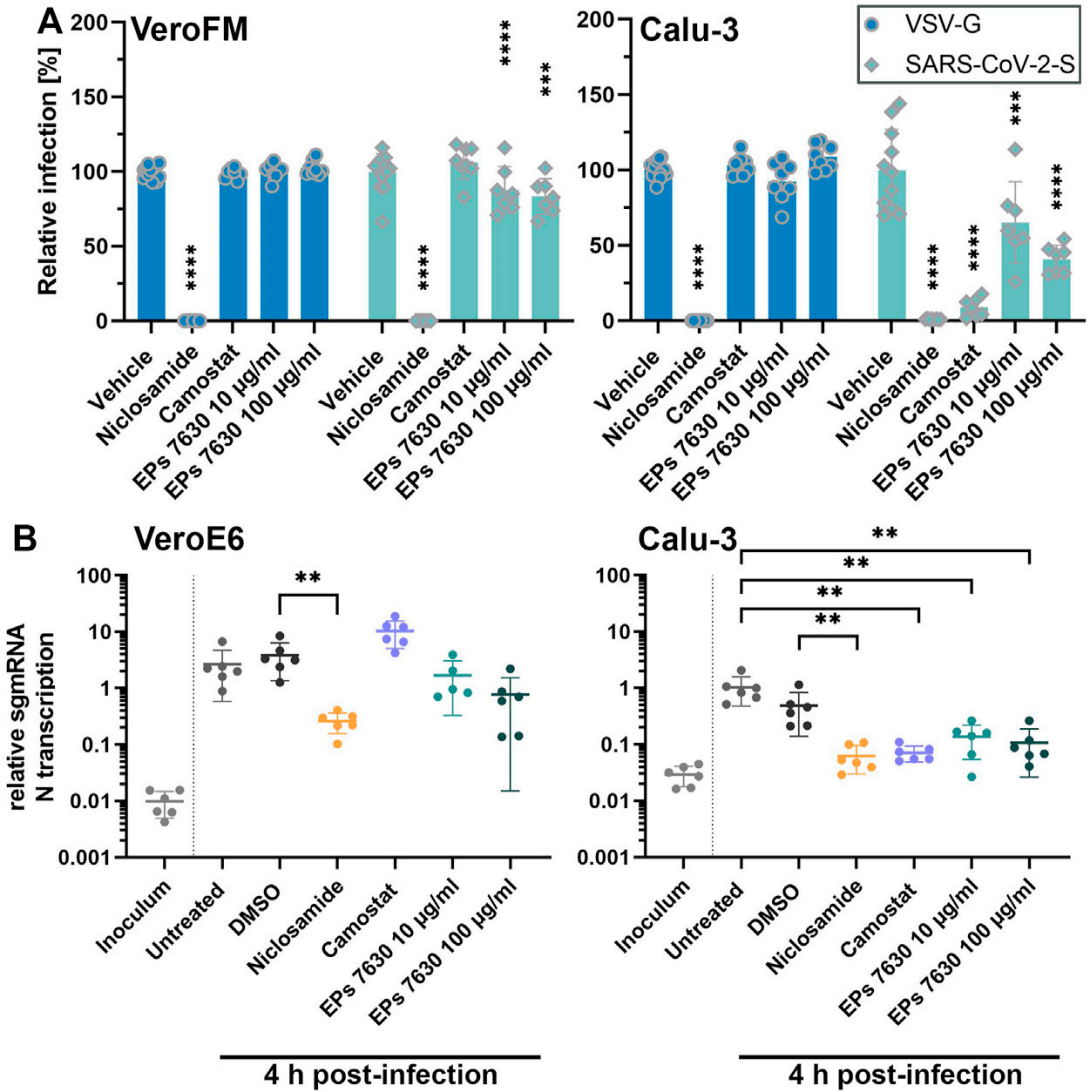
EPs 7630 inhibits entry of VSVpp-SARS-CoV-2-S and SARS-CoV-2 in TMPRSS2-negative VeroFM and TMPRSS2-positive Calu-3 cells. Cells were pre-treated with the indicated compounds for 2 h pre-infection at 37°C. **(A)** Infection with luciferase-producing VSVpp-SARS-CoV-2-Spike (SARS-CoV-2-S), VSV-G control, or pCG1 vector control was done in the presence of compounds for 30 min at 4°C at 300 × g followed by 1.5 h incubation at 37°C to achieve synchronous infection. The medium was then replaced by compound-containing DMEM as indicated. As controls, we applied 10 µM niclosamide (endosomal entry blocker) and 10 µM camostat mesylate (TMPRSS2 inhibitor). Cell lysates were prepared after 16 h (VeroFM) or 24 h (Calu-3) and luminescence was measured using a multi-mode 96-well plate reader. Bars represent mean values and SD from at least *n* = 7 biological samples from two independent experiments. Vehicle = DMSO-(niclosamide) or medium (remaining compounds). Statistical significance (treatment vs. control) is indicated by (*) as determined by two-way ANOVA with Dunnett’s multiple comparison testing. (*) = *p* < 0.05; (**) = *p* < 0.01; (***) = *p* < 0.001; (****) = *p* < 0.0001. **(B)** VeroE6 and Calu-3 cells were pretreated with the indicated compound 2 h as described in **(A)**. Infection with SARS-CoV-2 (MOI = 1) was done by incubation for 15 min at 4°C, followed by 30 min at 37°C to achieve synchronized infection. The medium was then replaced by compound-containing DMEM as indicated. As controls, we applied 10 µM niclosamide (endosomal entry blocker) and 10 µM camostat mesylate (TMPRSS2 inhibitor). Cell lysates were prepared 4 h post-infection and subgenomic viral mRNA (sgmRNA) was quantified by RT-qPCR. Data are presented as gene expression relative to the reference gene *TBP* from *n* = 6 biological samples from 3 experiments. Statistical significance is indicated by (*) as determined by unpaired t-test. (*) = *p* < 0.05; (**) = *p* < 0.01; (***) = *p* < 0.001; (****) = *p* < 0.0001. DMSO = Dimethyl sulfoxide (vehicle for niclosamide).

**FIGURE 6:**
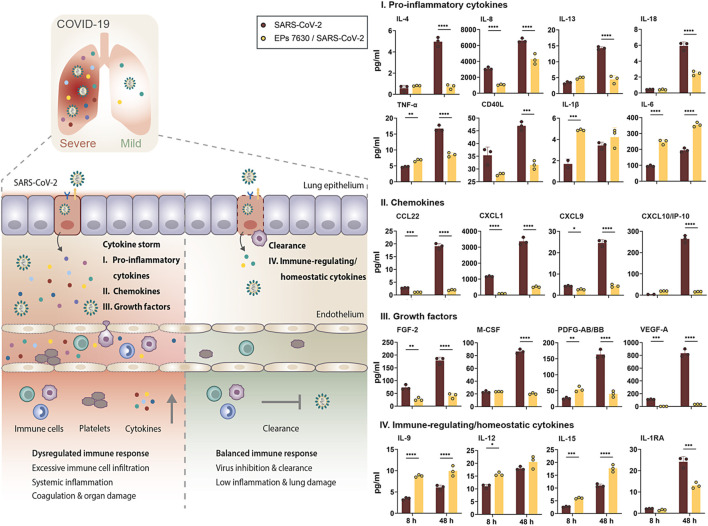
EPs 7630 treatment limits SARS-CoV-2-induced inflammation in Calu-3 lung cells. Supernatants from EPs 7630-treated (100 μg/ml) and EPs 7630-treated and SARS-CoV-2 infected (MOI = 0.0005) Calu-3 cells were analyzed at 8 and 48 h post-infection using the Human Cytokine/Chemokine/Growth Factor Multiplex Assay (Merck Millipore) with the Luminex MAGPIX System according to the manufacturer’s instructions. Data are derived from n = 3 biological samples (for IL-1β, CXCL10/IP10: *n* = 2–3). Vehicle = medium only. Statistical significance is indicated by (*) as determined by two-way ANOVA with Tukey’s multiple comparison test. Asterisks are shown only for significantly different data sets. (*) = *p* < 0.05; (**) = *p* < 0.01; (***) = *p* < 0.001; (****) = *p* < 0.0001.

In the original article, there was an error. The term “virus entry” was used instead of “virus propagation” as intended.

A correction has been made to the section “EPs 7630 and Distinct Fractions Limit Immune Gene Expression but Enhance Anti-inflammatory TNFAIP3 Induction,” paragraph 1.

“Apart from inhibiting virus entry, EPs 7630 has immunomodulatory effects (**Noldner and Schotz, 2007**; **Peric et al., 2021**) that might be beneficial for counteracting virus infections and preventing inflammation and immune dysregulation (cytokine storm), which is associated with high COVID-19 morbidity and mortality. The variable SARS-CoV-2 inhibition of the different EPs 7630 fractions (**Figure 4B**) encouraged us to compare immunomodulatory effects of EPs 7630 and its fractions in SARS-CoV-2-infected Calu-3 cells. Whereas EPs 7630 or its fractions (each 100 μg/ml) alone (**Figure 5**, green bar; **Supplementary Figure S7**) had no major effects on pro-inflammatory (*CCL5, IL6, IL1B*), IFN-dependent (*IFNB1, IFIT1, MX1*), or anti-inflammatory (*TNFAIP3*) gene expression, SARS-CoV-2 infection resulted in 10 to 100-fold increased expression of all genes except *TNFAIP3* at 48 h post-infection (**Figure 5**, red bars). EPs 7630 treatment post-SARS-CoV-2 infection resulted in a significant reduction of *IL1B* gene expression and strong upregulation of anti-inflammatory *TNFAIP3*. All other genes showed a limited but non-significant decrease in gene activation suggesting either limited transcriptional regulation or restoration of mRNA levels late in infection. Notably, reduced virus growth as a consequence of entry inhibition (see **Figures 2, 4**) might also generally limit the SARS-CoV-2-induced upregulation of immune genes. Still, the low molecular weight fractions <1 and 1–3 kDa, which had limited effects on virus propagation using 100 μg/ml (see **Figure 4B**), resembled EPs 7630-dependent gene regulation patterns with the strongest effects on *IL1B* and *TNFAIP3* during SARS-CoV-2 infection. The 4 fractions between 3 and >30 kDa had minor effects on anti-inflammatory *TNFAIP3*, but strong inhibitory effects on most of the pro-inflammatory and IFN-dependent genes. The differential gene activation patterns might be explained by the composition of the fractions containing gallocatechins, benzopyranones such as umckalin and umckalin sulfate, and purine derivatives in the two low molecular weight fractions, and increasing amounts of di-, tri-, hexa-, oligo-, and polymeric prodelphinidins in the high molecular weight fractions (**Table 1**).”

In the original article, there was a mistake in the legend for Figures 2, **4**–6 as published. A copy-paste error was detected for p value definitions (definition for “****” is accidentally labeled with “*”). The correct legends for Figure 2 and Figure 6 appear in the new figures. The correct legends for **Figure 4** and **Figure 5** appear below.

**FIGURE 4** | EPs 7630 molecular fractions differentially inhibit SARS-CoV-2 propagation. **(A)** Infection of Calu-3 cells with SARS-CoV-2-Spike VSVpp (SARS-CoV-2-S) or VSV-G as control was done in the presence of compounds for 30 min at 4°C at 500 × g followed by 1-h incubation at 37°C. Cell lysates were prepared after 24 h and the luciferase signal was measured using a multi-mode 96-well plate reader. Bars represent mean values and SD from n = 5–8 biological samples from two independent experiments. Technical outliers were removed from the analysis. **(B)** Calu-3 cells were infected with SARS-CoV-2 (MOI = 0.0005) and treated with fractions of ultrafiltrated EPs 7630 simultaneously. Virus-containing supernatants were collected 48 h post-infection and viral titers were determined as plaque-forming units (PFU)/ml by plaque titration assay. Data are derived from *n* = 3 biological samples (for <30 kDa 66 μg/ml, 5–10 kDa 33 μg/ml, 1–3 kDa 33 μg/ml, and <1 kDa 33 μg/ml: *n* = 2). Vehicle = medium. Statistical significance (treatment vs. vehicle) is indicated by (*) as determined by two-way **(A)** or one-way **(B)** ANOVA with Dunnett’s multiple comparison testing. Asterisks are shown only for significantly different data sets in comparison to vehicle treatment. (*) = *p* < 0.05; (**) = *p* < 0.01; (***) = *p* < 0.001; (****) = *p* < 0.0001.

**FIGURE 5** | Calu-3 cells treated with EPs 7630 or ultrafiltrated fractions show enhanced anti-inflammatory responses during SARS-CoV-2 infection. Calu-3 cells were treated with EPs 7630 (100 μg/ml), infected with SARS-CoV-2 (MOI = 0.0005; vehicle), or a combination of both. Additionally, cells were treated with ultrafiltrated fractions of EPs 7630 and infected with SARS-CoV-2. Cell lysates were prepared 48 h post-infection and cellular RNA of the indicated immune genes was quantified by RT-qPCR. Green bars show EPs 7630-treated, non-infected cells for comparison. Vehicle = medium. Data are derived from *n* = 3 biological samples and are presented as fold gene expression relative to untreated cells and normalized to reference gene expression (*TBP*). Statistical significance in samples from SARS-CoV-2 infected cells (treatment vs. vehicle) is indicated by (*) as determined by two-way ANOVA with Dunnett’s multiple comparison testing. (*) = *p* < 0.05; (**) = *p* < 0.01; (***) = *p* < 0.001; (****) = *p* < 0.0001.

The authors apologize for this error and state that this does not change the scientific conclusions of the article in any way. The original article has been updated.

