# Supplementary material for: Antiviral and Immunomodulatory Effects of Pelargonium sidoides DC. Root Extract EPs® 7630 in SARS-CoV-2-Infected Human Lung Cells
Source: Front Pharmacol. 2021 Oct 25;12:757666. doi: 10.3389/fphar.2021.757666 (PMC8573200; doi:10.3389/fphar.2021.757666)
Supplement: Supplementary file 1 [file DataSheet1.PDF]

Supplementary Material

1 Supplementary Figures and Tables

1.1 Supplementary Figures

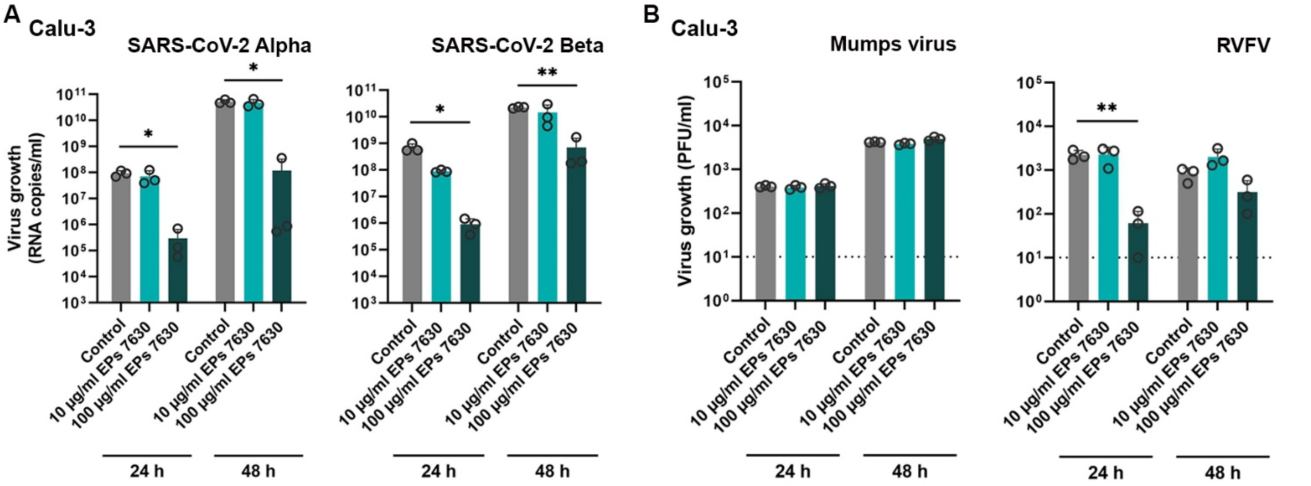

Supplementary Figure 1. Differential inhibition of representative RNA viruses by EPs 7630. (A) Growth comparison of SARS-CoV-2 variants of concern Alpha and Beta in Calu-3 cells using an MOI of 0.0005 and indicated concentrations of EPs 7630. Virus-containing supernatants were collected 24 h and 48 h post-infection and viral RNA levels were determined by RT-qPCR targeting the viral N gene. Bars represent mean values and SD from n=3 biological samples. Statistical significance (treatment vs. control) is indicated by (*) as determined by 2-way ANOVA multiple comparison testing. (*) = p<0.05; (**) = p<0.01. Control = medium only. **(B)** Comparison of virus growth of Mumps virus (genotype G) and Rift Valley fever virus (RVFV clone 13) in Calu-3 cells using an MOI of 0.01 and indicated concentrations of EPs 7630. Virus-containing supernatants were collected 24 and 48 h post-infection and viral titers were determined as plaque-forming units (PFU)/ml by plaque titration assay. Bars represent mean values and SD from n=3 biological samples. Statistical significance (treatment vs control) is indicated by (*) as determined by 2-way ANOVA with Tukey's multiple comparison testing. (**) = p<0.01. Control = medium only.

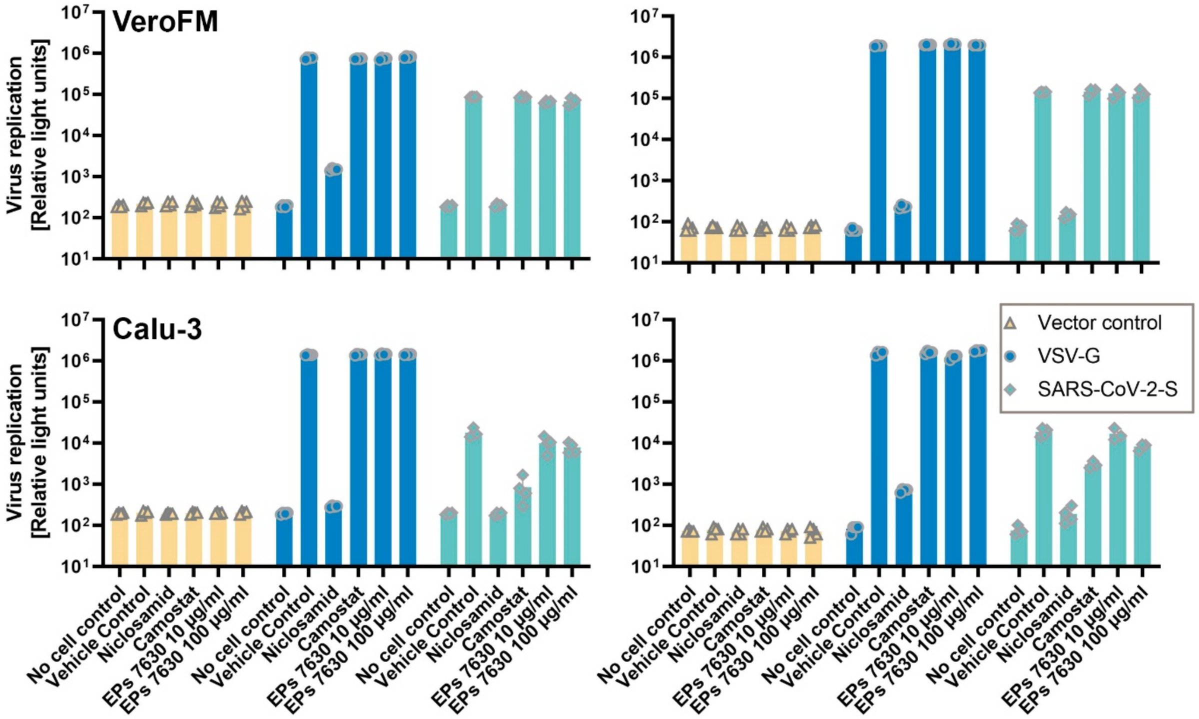

Supplementary Figure 2. Unprocessed data from VSV-pseudo-particle-based entry experiments in VeroFM and Calu-3 cells. Cells were pre-treated with the indicated compounds for 2 hours pre-infection at 37°C. Infection with SARS-CoV-2-Spike VSVpp (SARS-CoV-2-S), VSV-G control, or vector control (pcg1 vector backbone) was done in the presence of compounds or controls for 30 minutes at 4°C at 500 x g followed by 1-hour incubation at 37°C. As controls, we applied niclosamide (endosomal entry blocker) and camostat mesylate (TMPRSS2 inhibitor). Cell lysates were prepared after 16 h (VeroFM) or 24 h (Calu-3) and the luciferase signal was measured using a multi-mode 96-well plate reader. Bars represent mean values and SD from n=4 biological samples. Experimental data were verified in at least one biologically independent experiment with n=4 (right panels). Control=DMSO-(Camostat) or medium (remaining compounds). No cell control=medium only.

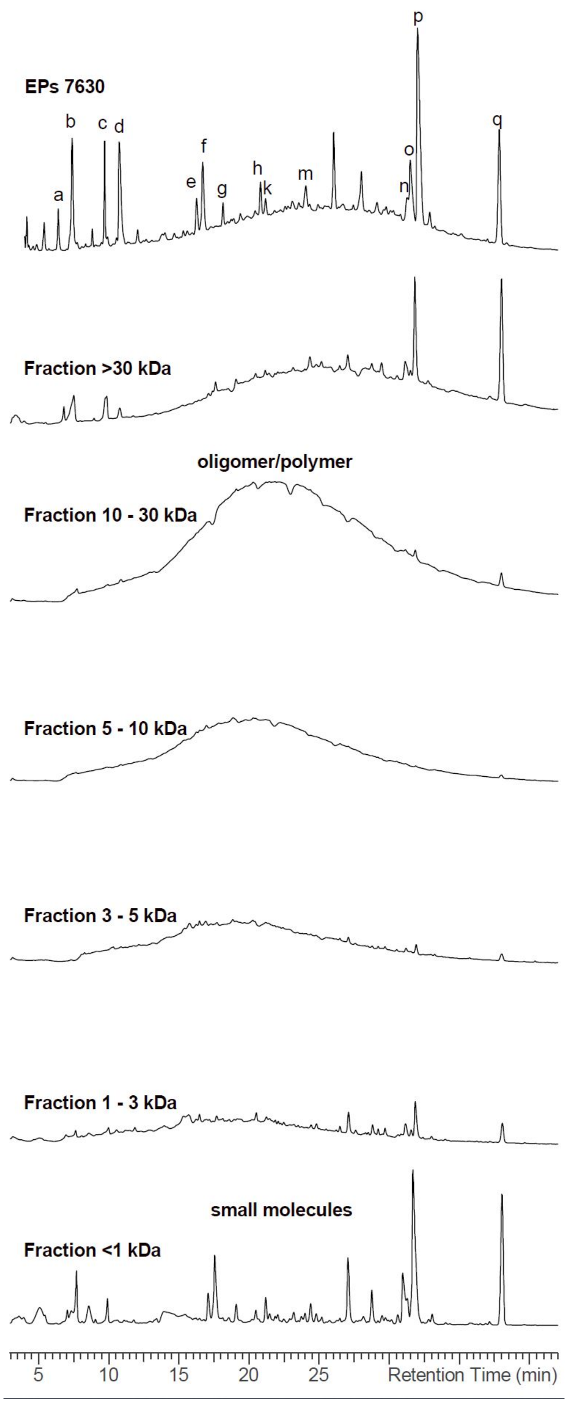

Supplementary Figure 3. Labeled HPLC-UV chromatograms of the fractions at 280 nm. The small molecule assignments a – q are described in **Supplementary Table 2**. In the fraction >30 kDa the polymeric prodelphinidins appear as a broad signal between 15 – 40 min. Umckalin sulfate and umckalin (p and q, respectively) can also be detected in this fraction. In the fractions 1 – 30 kDa, oligomeric prodelphinidins appear as broad signals between 10 – 40 min. In the fraction <1 kDa, only small molecules are detectable, with no broad absorption of prodelphinidins.

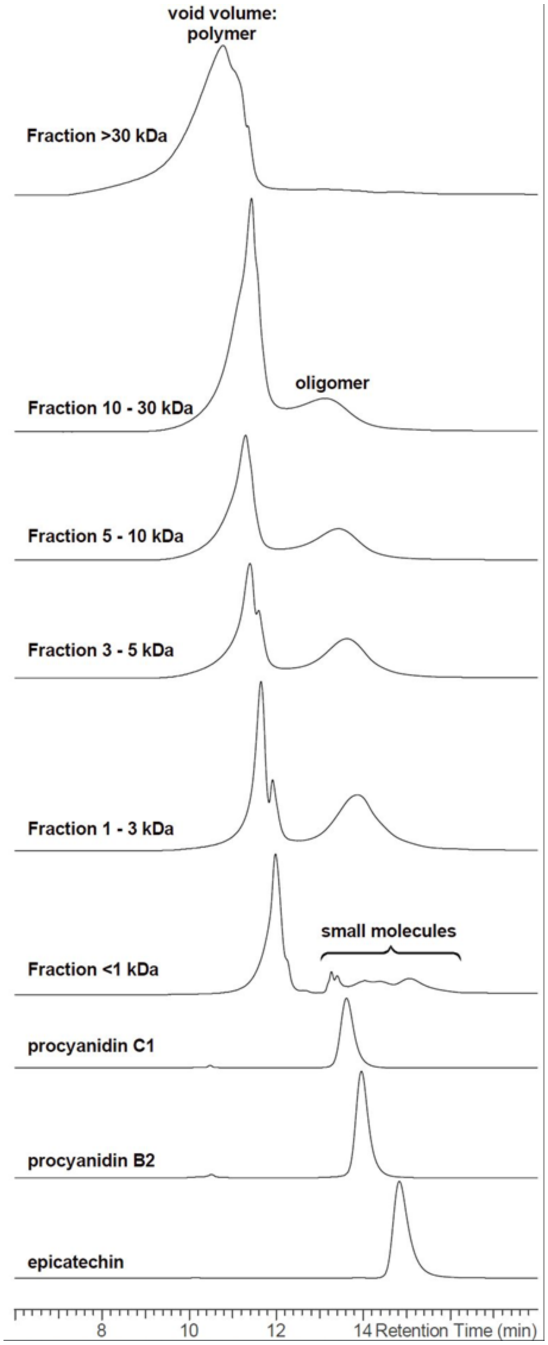

Supplementary Figure 4. GPC-UV chromatograms of the fractions at 230 nm. For estimation of the molecular size of prodelphinidins in the fractions, the elution times were calibrated using epicatechin (monomer), procyanidin B2 (dimer), and procyanidin C1 (trimer) as model substances of proanthocyanidins. In the fraction >30 kDa only polymeric prodelphinidins are detectable as an absorption in the void volume. In the fractions 1 – 30 kDa, the oligomerization degree of oligomeric prodelphinidins can be estimated considering the calibration by the model substances. In the fraction <1 kDa, small molecules are detectable as a broad set of signals between 13 – 16 minutes. Interestingly, intense signals between 11 – 12 minutes appear in all fractions. Since the molecular size of substances behind these signals may be too high as estimated from the fractionation protocol, these signals may be attributed to non-covalent aggregation or quaternary structures of prodelphinidins.

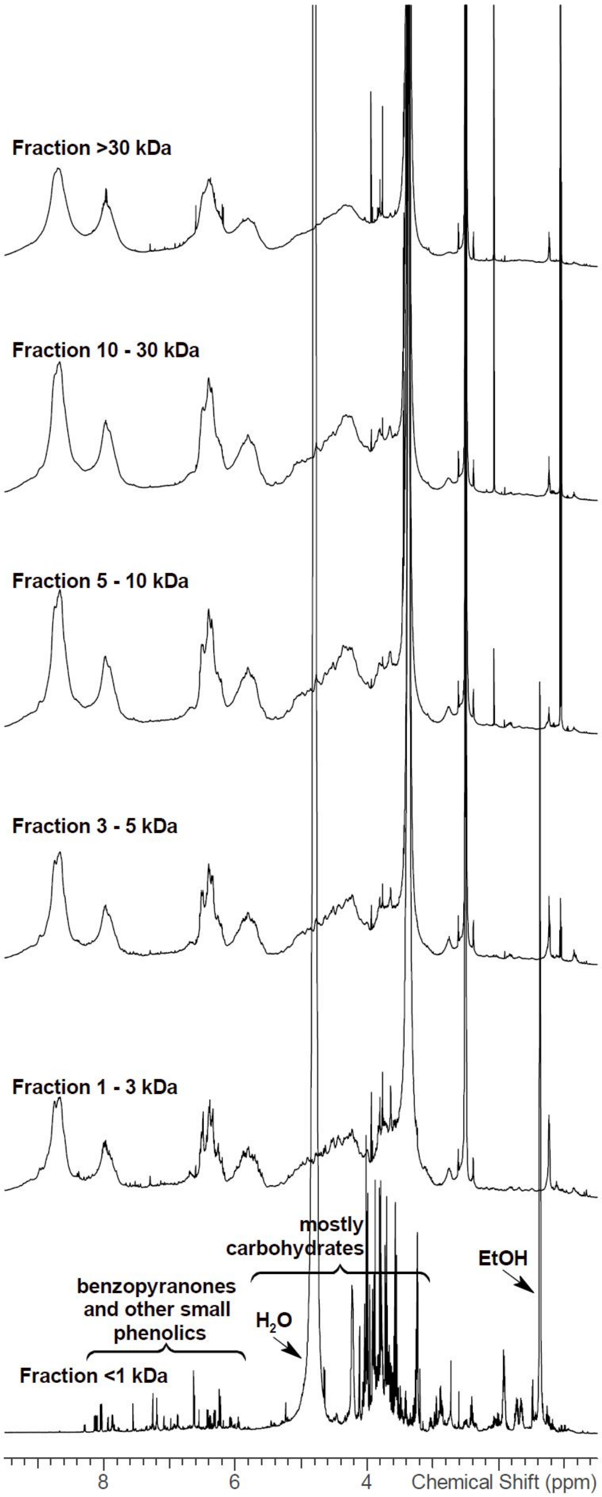

Supplementary Figure 5. ^{1}H-1D-NMR spectra of the fractions. In the fraction <1 kDa, only sharp signals of small molecules are detectable. The integral ratio of signal patterns of carbohydrates and small phenolics corresponds to approximately 5 to 1, respectively. The higher the molecular weight of substances in the fractions, the broader the signals become. This is consistent with increasingly faster relaxation times for bigger molecules induced by increasingly efficient dipolar relaxation correlating to slower molecular tumbling rates.

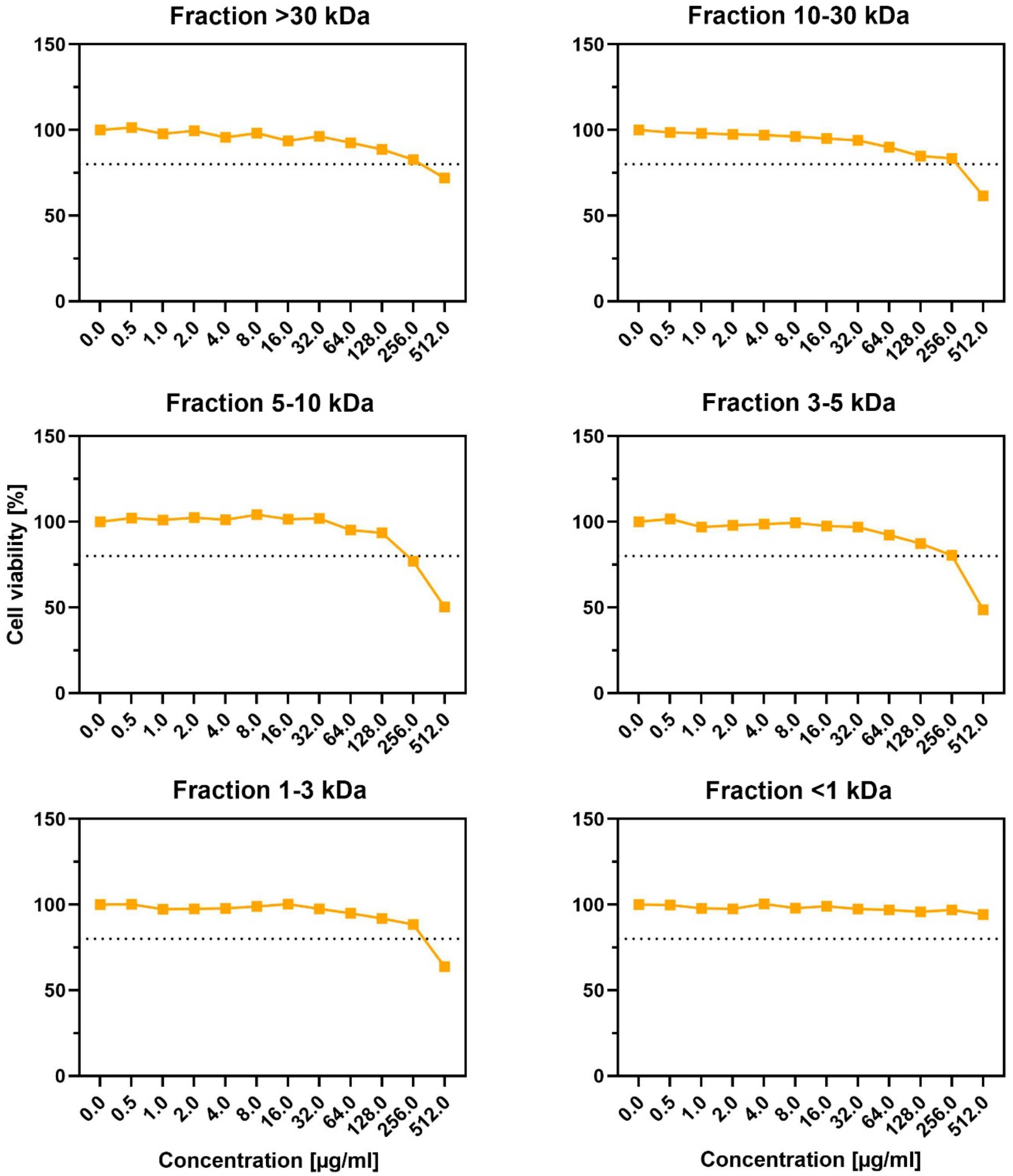

Supplementary Figure 6. Cell viability assay in Calu-3 cells for the different EPs 7630-derived ultrafiltration fractions. Calu-3 cells were treated with the indicated compound concentrations ranging from 0.5 to 512 µg/ml. A CellTiter-Glo® 2.0 Cell Viability Assay (Promega) was done 48 hours post-treatment. Viability threshold was set to 80%. The area below the dotted line indicates the range of experiments that are shown in the manuscript. Data are presented as percent of untreated cells (mean and SD) derived from n=3 biological samples.

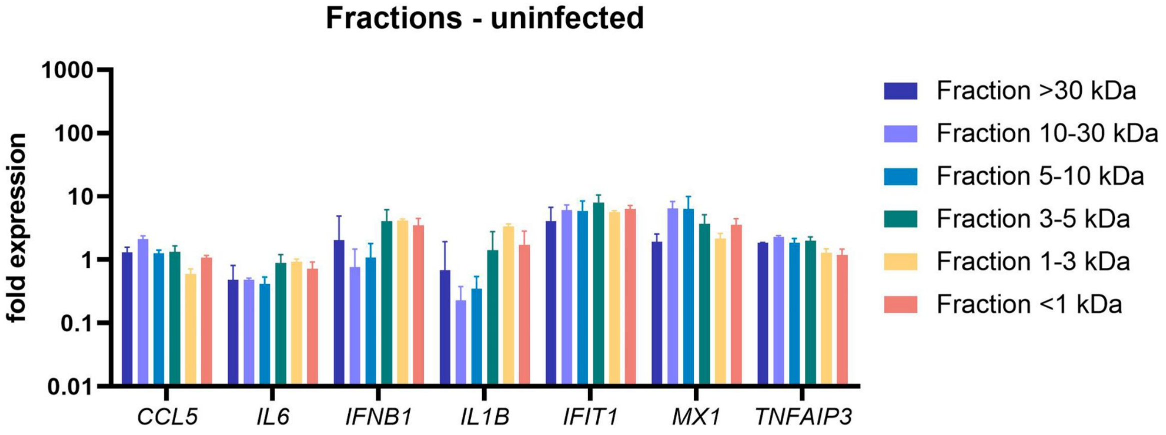

Supplementary Figure 7. Immune gene regulation by EPs 7630-derived ultrafiltration fractions. Calu-3 cells were treated with the indicated fractions of EPs 7630 (100 µg/ml). Cell lysates were prepared 48 h post-treatment and cellular RNA of the indicated immune genes was quantified by RT-qPCR. Data are derived from n= 3 biological samples and are presented as fold gene expression relative to untreated cells and normalized to reference gene expression (*TBP*).

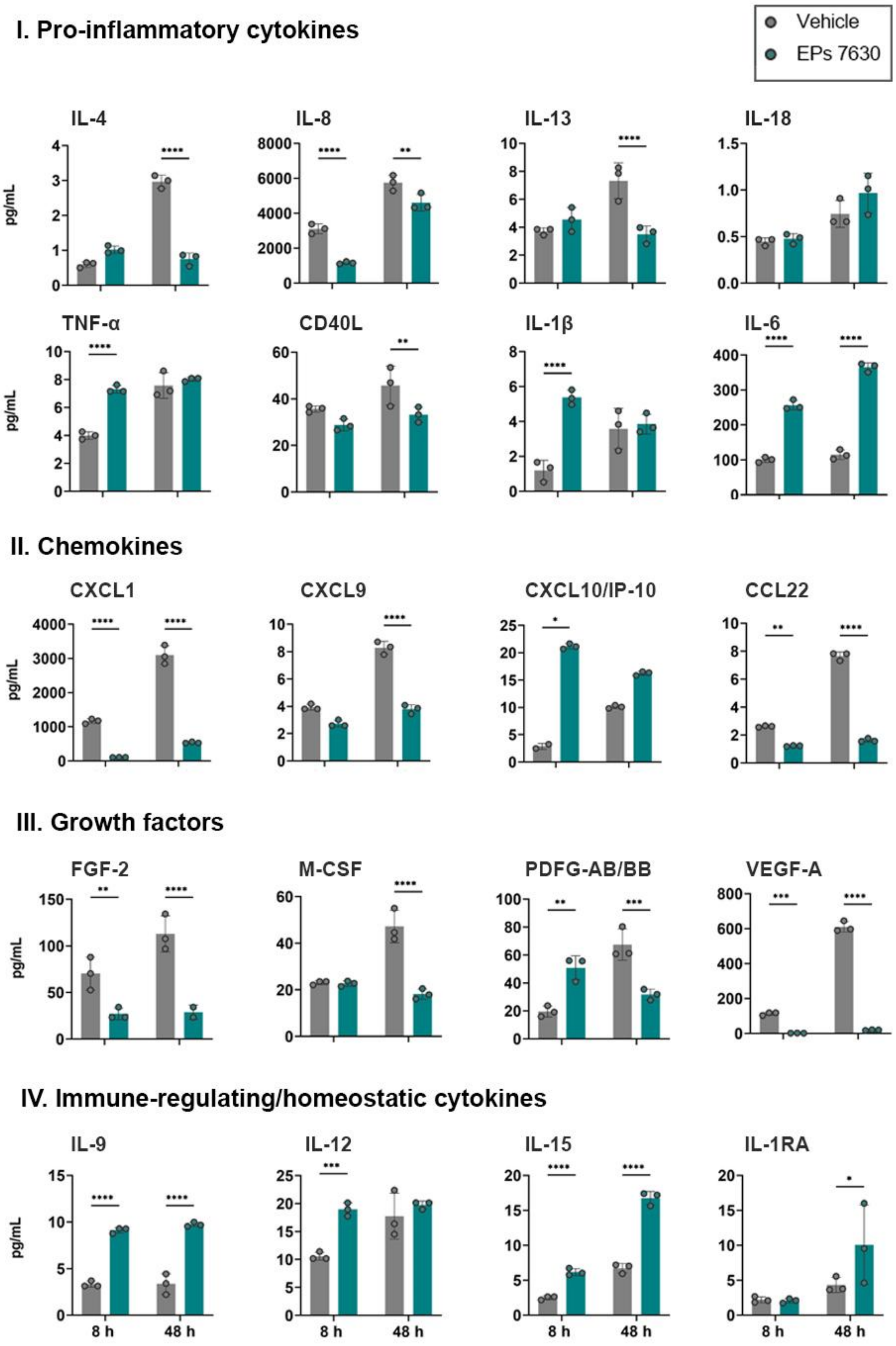
I. Pro-inflammatory cytokines
Vehicle
EPs 7630
IL-4
IL-8
IL-13
IL-18
TNF-α
CD40L
IL-1β
IL-6
pg/mL
II. Chemokines
CXCL1
CXCL9
CXCL10/IP-10
CCL22
III. Growth factors
FGF-2
M-CSF
PDFG-AB/BB
VEGF-A
IV. Immune-regulating/homeostatic cytokines
IL-9
IL-12
IL-15
IL-1RA
8 h
48 h

Supplementary Figure 8. EPs 7630-induced cytokine production Supernatants from EPs 7630-treated (100 µg/ml) and vehicle (DMEM)-treated Calu-3 cells were analyzed using the Human Cytokine/Chemokine/Growth Factor Multiplex Assay (Merck Millipore) with the Luminex MAGPIX System according to the manufacturer's instructions. Data are derived from n=3 biological samples (for CXCL10/IP10, FGF2: n=2-3). Statistical significance is indicated by (*) as determined by two-way ANOVA with Tukey's multiple comparison test. Asterisks are shown only for significantly different data sets. (*) = $p<0.05$; (**) = $p<0.01$; (***) = $p<0.001$; (*) = $p<0.0001$.

1.2 Supplementary Tables

Supplementary Table 1. Oligonucleotides for RT-qPCR analysis

Name	Sequence	Source
CCL5		
h010_CCL5_F	TGCCCACATCAAGGAGTATTTC	(Sima et al., 2018)
h010_CCL5_p	TCACCCGAAAGAACCGCCAAGT	
h010_CCL5_R	CCATCCTAGCTCATCTCCAAAG	(Sima et al., 2018)
IL1B		
hIL1B_f	ACGCTCCGGGACTCACAG	In-house
hIL1B_p	AAGCTCTCCACCTCCAGGGACAGG	In-house
hIL1B_r	CAAAGGACATGGAGAACACC	In-house
IL6		
hIL6_f	GGATTCAATGAGGAGACTTGC	In-house
hIL6_p	AATCATCACTGGTCTTTTGGAGTTTGAGG	In-house
hIL6_r	CACAGCTCTGGCTTGTTCC	In-house
IFIT1		
#292 huISG56 F	CCTGGAGTACTATGAGCGGGC	(Holzinger et al., 2007)
#293 huISG56 P	ACAGAGTTCTCAAAGTCAGCAGCCAGTCTCAGG	(Holzinger et al., 2007)
#294 huISG56 R	TGGGTGCCTAAGGACCTTGTC	(Holzinger et al., 2007)
IFNB1		

hIFNb1_f	AGGATTCTGCATTACCTGAAGG	(Holzinger et al., 2007)
hIFNb1_p	TCCACTCTGACTATGGTCCAGGCA	(Holzinger et al., 2007)
hIFNb1_r	GGCTAGGAGATCTTCAGTTTCG	(Holzinger et al., 2007)
MX1		
huMX1 F Q298	CGGATGCTTCAGAGGTAGAAAAG	In-house
huMX1 P Q299	AAATTAATAAAGCCCAGAATRCCATYGCC	In-house
huMX1 R Q300	CTCATGACTGATTCCCATTCCTT	In-house
TBP		
qRT_hTBP_fwd #128	GCTGCGGTAATCATGAGGATAAG	(Biesold et al., 2011)
qRT_hTBP_prb #129	AGCCACGAACCACGGCACTGATTTT	(Biesold et al., 2011)
qRT_hTBP_rev #130	TGCACACCATTTTCCCAGAA	(Biesold et al., 2011)
TNFAIP3		
panTNFAIP3_f	CTCAACTGGTGTCGAGAAGTC	In-house
panTNFAIP3_p	TGAACRCCCCACATGTACTGAGAAG	In-house
panTNFAIP3_r	GCCAGCGGAATTTAAAGTTGC	In-house

Supplementary Table 2. Assignment of HPLC signals of EPs 7630 from Supplementary Figure 3.

Assigned Peak in Supplementary Figure 5	*Name*				
a	Adenosine 3′,5′-cyclic monophosphate				
b	Guanosine 3′,5′-cyclic monophosphate				
c	1-Methylguanosine 3′,5′-cyclic monophosphate				
g	gallocatechin				
k	epigallocatechin				
Benzopyranones	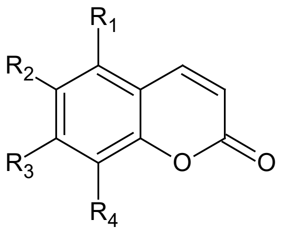	R_1	R_2	R_3	R_4
d	6,8-bissulfooxy-7-hydroxy-2H-1-benzopyran-2-one	H	SO_4^-	OH	SO_4^-
e	6,7-dihydroxy-8sulfooxy-2H-1-benzopyran-2-one	H	OH	OH	SO_4^-
f	7,8-dihydroxy-6-sulfooxy-2H-1-benzopyran-2-one	H	SO_4^-	OH	OH
h	8-hydroxy-7-methoxy-6-(sulfooxy)-2H-1-benzopyran-2-one	H	SO_4^-	OCH_3	OH
m	6-methoxy-7-sulfooxy-2H-1-benzopyran-2-one	H	OCH_3	SO_4^-	H

n	5,6-dimethoxy-7,8-dihydroxy-2H-1-benzopyran-2-one	OCH_3	OCH_3	OH	OH
o	7-hydroxy-5,6-dimethoxy-8-sulfooxy-2H-1-benzopyran-2-one	OCH_3	OCH_3	OH	SO_4^-
p	5,6-dimethoxy-7-sulfooxy-2H-1-benzopyran-2-one (umckalin-7-sulfate)	OCH_3	OCH_3	SO_4^-	H
q	7-hydroxy-5,6-dimethoxy-2H-1-benzopyran-2-one (umckalin)	OCH_3	OCH_3	OH	H

1.3 References

Biesold, S.E., Ritz, D., Gloza-Rausch, F., Wollny, R., Drexler, J.F., Corman, V.M., et al. (2011). Type I Interferon Reaction to Viral Infection in Interferon-Competent, Immortalized Cell Lines from the African Fruit Bat Eidolon helvum. *Plos One* 6(11). ARTN e28131 doi: 10.1371/journal.pone.0028131.

Holzinger, D., Jorns, C., Stertz, S., Boisson-Dupuis, S., Thimme, R., Weidmann, M., et al. (2007). Induction of MxA gene expression by influenza A virus requires type I or type III interferon signaling. *J Virol* 81(14)**,** 7776-7785. doi: 10.1128/JVI.00546-06.

Sima, J., Yan, Z., Chen, Y., Lehrmann, E., Zhang, Y., Nagaraja, R., et al. (2018). Eda-activated RelB recruits an SWI/SNF (BAF) chromatin-remodeling complex and initiates gene transcription in skin appendage formation. *Proc Natl Acad Sci U S A* 115(32)**,** 8173-8178. doi: 10.1073/pnas.1800930115.
